# Venus Kinase Receptors Control Reproduction in the Platyhelminth Parasite *Schistosoma mansoni*

**DOI:** 10.1371/journal.ppat.1004138

**Published:** 2014-05-29

**Authors:** Mathieu Vanderstraete, Nadège Gouignard, Katia Cailliau, Marion Morel, Steffen Hahnel, Silke Leutner, Svenja Beckmann, Christoph G. Grevelding, Colette Dissous

**Affiliations:** 1 Center for Infection and Immunity of Lille, Inserm U1019, CNRS-UMR 8204, University Lille Nord de France, Institut Pasteur de Lille, Lille, France; 2 EA 4479, IFR 147, Universite Lille 1 Sciences et Technologies, Villeneuve d'Ascq, France; 3 Institute for Parasitology, Justus-Liebig-University Giessen, Giessen, Germany; Uniformed Services University, United States of America

## Abstract

The Venus Kinase Receptor (VKR) is a single transmembrane molecule composed of an intracellular tyrosine kinase domain close to that of insulin receptor and an extracellular Venus Flytrap (VFT) structure similar to the ligand binding domain of many class C G Protein Coupled Receptors. This receptor tyrosine kinase (RTK) was first discovered in the platyhelminth parasite *Schistosoma mansoni*, then in a large variety of invertebrates. A single *vkr* gene is found in most genomes, except in *S. mansoni* in which two genes *Smvkr1* and *Smvkr2* exist. VKRs form a unique family of RTKs present only in invertebrates and their biological functions are still to be discovered. In this work, we show that SmVKRs are expressed in the reproductive organs of *S. mansoni*, particularly in the ovaries of female worms. By transcriptional analyses evidence was obtained that both SmVKRs fulfill different roles during oocyte maturation. Suppression of *Smvkr* expression by RNA interference induced spectacular morphological changes in female worms with a strong disorganization of the ovary, which was dominated by the presence of primary oocytes, and a defect of egg formation. Following expression in *Xenopus* oocytes, SmVKR1 and SmVKR2 receptors were shown to be activated by distinct ligands which are L-Arginine and calcium ions, respectively. Signalling analysis in *Xenopus* oocytes revealed the capacity of SmVKRs to activate the PI3K/Akt/p70S6K and Erk MAPK pathways involved in cellular growth and proliferation. Additionally, SmVKR1 induced phosphorylation of JNK (c-Jun N-terminal kinase). Activation of JNK by SmVKR1 was supported by the results of yeast two-hybrid experiments identifying several components of the JNK pathway as specific interacting partners of SmVKR1. In conclusion, these results demonstrate the functions of SmVKR in gametogenesis, and particularly in oogenesis and egg formation. By eliciting signalling pathways potentially involved in oocyte proliferation, growth and migration, these receptors control parasite reproduction and can therefore be considered as potential targets for anti-schistosome therapies.

## Author Summary

Schistosomiasis is a chronic, debilitating disease affecting more than 200 million people in the world caused by parasitic flatworms of the genus *Schistosoma*. Pathology is mainly due to massive egg production by parasites and formation of granulomas around the eggs trapped in liver and different organs. Therefore, targeting the molecular processes responsible for gonad development or egg production in schistosomes appears as a valuable strategy to reduce pathogenesis and dissemination of schistosomiasis. In the present study, we investigated the importance of Venus Kinase Receptors (VKRs) which are unusual receptor tyrosine kinases (RTKs) with an extracellular Venus Flytrap (VFT) ligand-binding domain in the control of reproduction of schistosomes. SmVKRs are expressed in female ovaries of *Schistosoma mansoni* and the knock-down of their expression provoked dramatic alterations of the oocyte content in ovaries and reduction of egg formation. SmVKRs were also shown to activate different signalling pathways potentially involved in oocyte proliferation, growth and migration. Therefore our results demonstrate that VKRs are essential actors of oogenesis and egg formation in *S. mansoni*. Moreover, their presence in a large variety of invertebrate species including other helminth parasites and insect parasite vectors can open new perspectives in the control of various vector-borne infectious diseases.

## Introduction

Trematode parasites of the *Schistosoma* genus are responsible for schistosomiasis or bilharzia, one of the most important parasitic endemias worldwide in terms of mortality and morbidity. According to the World Health Organisation, more than 240 million people are currently infected by schistosomes, with about 200 000 deaths per year [1].

The pathology of schistosomiasis mostly results from the accumulation of parasite eggs in host tissues. Indeed, among the several hundreds of eggs laid daily by each female schistosome, a large part gets trapped into host tissues and elicits immune responses, such as inflammation and granuloma formation, causing severe disorders, particularly hepatosplenomegaly, hepatic fibrosis and bladder cancer [2]. Praziquantel (PZQ) is the only drug currently used to cure schistosomiasis. This drug is active against the three main species infecting humans (*S. mansoni, S. haematobium, S. japonicum*). However, its widespread use for mass treatment since the early 80's, has already led to the emergence/of drug-insensitive *Schistosoma* strains. Moreover, a limit of PZQ is that it does not affect the larval parasites and, therefore, does not provide a total clearance of the infection [3]–[6]. In the absence of a vaccine much efforts are currently made to characterize molecules that control survival, growth and reproduction of schistosomes in order to identify targets for novel drugs against these parasites [7]–[9].

In this context, several schistosome protein kinases (PK) have been studied during the last decade and some of them were shown to be involved in gametogenesis and egg formation in the parasite *S. mansoni* [10]–[20]. Among these kinases are cellular PKs such as Polo-like kinases (Plks), which regulate cell-cycle progression during M-phase by activating cyclin-dependent PKs. Two Plks, SmPlk1 and SmSak, have been characterized in *S. mansoni* and their role in the control of parasite reproduction has been demonstrated [17]–[19]. Besides these mitotic kinases, tyrosine kinases (TKs) were also shown to play essential roles in schistosome reproduction [11], [16]. TKs constitute a large family of receptor and cytosolic molecules that regulate development, cell division, differentiation and metabolism in many organisms, and they actually represent major targets in drug discovery programs against cancer and metabolic disorders [21], [22]. Recent work indicated that *S. mansoni* TKs can be as well considered as interesting targets against schistosomes. By inhibiting the kinase activities of schistosome Src, Abl, and Syk cytosolic TKs with the commercial TK inhibitors Herbimycin, Imatinib, Piceatannol, respectively, dramatic changes were observed in the reproductive organs of *in vitro*-cultured adult *S.mansoni*, affecting gametogenesis and egg production [11]–[16]. Also, the harmful effect on schistosomes of other TK inhibitors (tyrphostins) directed against human EGF and insulin receptors revealed the importance of RTK signalling in major processes of embryogenesis and reproduction in these parasites [23]–[25].

Few years ago, we discovered in *S. mansoni* an atypical receptor tyrosine kinase (RTK) named Venus Kinase Receptor [26]. VKR is a transmembrane molecule composed of an intracellular TK domain close to that of Insulin Receptors (IRs), and an extracellular ligand binding domain (LBD) with a Venus Flytrap (VFT) structure similar to that of many class C G Protein Coupled Receptors (GPCR) [27]. Such a VFT-TK fusion was described for the first time in *S. mansoni*, but recent analysis of genomic data has allowed us to extend the presence of VKRs to five bilaterian phyla (Platyhelminthes, Arthropoda, Annelida, Mollusca, Echinodermata) as well as to the Cnidaria phylum [28], [29]. We showed that VKR kinase activity is inducible upon binding of extracellular amino-acids and molecular modeling of the VFT domain confirmed the structure of the conserved amino-acid binding site [28]. Preliminary experiments indicated that VKRs were preferentially expressed in larvae and in gonads of several species [28], suggesting a role in development and reproduction. A single *vkr* gene was usually found in each invertebrate genome but exceptionally the genome of *S. mansoni*, as that of another trematode *Clonorchis*, contains two genes *vkr1* and *vkr2* with a similar organization but encoding distinct proteins [29], [30]. The expression of *Smvkr1* and *Smvkr2* was shown to be variable in the different parasitic stages, likely indicating their independent regulation [30].

In this work, we investigated the function of SmVKR in the reproduction of schistosomes. We first showed that *Smvkr1* and *Smvkr2* are expressed in adult female organs at different sites, and the results of RNAi experiments suggest that SmVKR1 and SmVKR2 participate in major pathways along the maturation of oocytes in female schistosomes. The two VKRs are activated by distinct ligands, and the search for interacting partners by yeast two-hybrid screening coupled to the analysis of signalling pathways induced by SmVKRs in *Xenopus* oocytes support the hypothesis that SmVKR1 and SmVKR2 exert different roles in gametogenesis and reproduction processes in schistosomes.

## Results

### SmVKR1 and SmVKR2 are expressed in female reproductive organs

**Figure 1:**
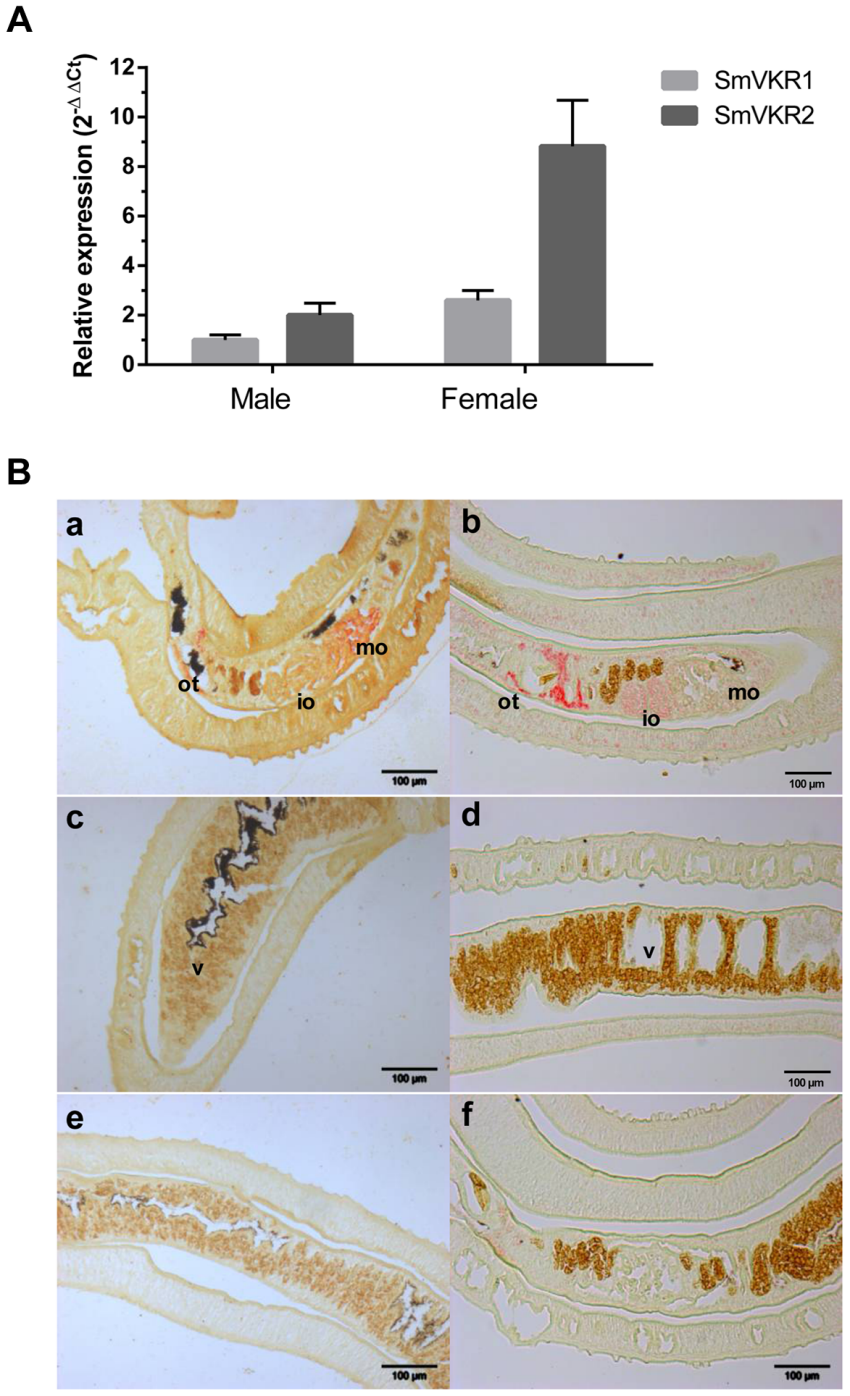
SmVKR1 and SmVKR2 expression patterns in adult worms. A: Quantification of *Smvkr1* and *Smvkr2* transcripts in adult female and male worms by quantitative RT-PCR. Tubulin transcripts were used as internal controls for each condition. For graphical representation, values were expressed as relative fold-difference using the ΔΔCt method and the *Smvkr1* ΔCt value in males as reference. Values are expressed as the mean (+/- SEM) of three determinations. B: Localization in sections of paired adult worms of *Smvkr1* (a, c, e) and *Smvkr2* (b, d, f) transcripts by *in situ* hybridization. *Smvkr1* transcripts were detected in mature oocytes (a) whereas *Smvkr2* transcripts were detected in immature oocytes and in the area surrounding the ootype (b). No expression of *Smvkr1* (c) and *Smvkr2* (d) was detected in the vitellarium. Sense probes of *Smvkr1* and *Smvkr2* were used respectively as controls in e and f. Abbreviations: io: immature oocytes, mo: mature oocytes, ot: ootype, v: vitellarium. Scale bar: 100µm.

We have shown previously that SmVKRs were expressed in all developmental stages of the parasite *S. mansoni* and that the expression of SmVKR2 was globally higher than that of SmVKR1, except in the sporocyst stage [30]. Using as a reference α-tubulin transcripts, we show here that both *Smvkr1* and *Smvkr2* transcripts are more abundant in female than in male adult worms, and we confirm that *Smvkr2* is transcribed more actively than *Smvkr1* in each gender (Figure 1,A). Former studies have already indicated that SmVKR1 (firstly named SmRTK-1) was expressed in the parenchyma of male worms and in the ovary of female worms [26]. In this work, we have localized *Smvkr1* and *Smvkr2* transcripts on sections of adult worm pairs by *in situ* hybridization. Results (Figure 1,B) demonstrate the presence of abundant *Smvkr1* transcripts in the female ovary. From the staining intensity, indications were obtained for a more intense staining of the big, mature oocytes which are contained in the posterior part of the ovary whereas the immature oocytes (oogonia) are located within the smaller, anterior part of the ovary. *Smvkr1* labelling is also observed around the ootype (a female structure which receives fertilized oocytes and vitelline cells, and constitutes the egg-forming organ) and in the parenchyma of males. *Smvkr2* transcripts are found also in the ovary but, unlike *Smvkr1* transcripts, their abundance seemed to be higher within the anterior part of the ovary containing immature oocytes. An intense *Smvkr2* labelling was also obtained in the region of the ootype and its surrounding area, which could correspond to the Mehlis gland and/or the oviduct (Figure 1,B). In male testes, a specific labelling could be observed for *Smvkr1* as well as for *Smvkr2*, which was more evident following longer exposure (Figure S1).

Hahnel et *al* [31] have recently established a protocol to isolate schistosome reproductive organs usable for morphological and structural studies, and as sources of proteins and RNA. Q-PCR experiments performed with RNA extracted from isolated ovaries have shown that *Smvkr1* and *Smvkr2* are expressed at the same level in the ovaries of sexually mature females recovered from bisexual parasite infections (Figure S2,A). However, within the ovaries isolated from female worms grown in the absence of males and which are known to contain only immature and undifferentiated cells (oogonia) [32], we observed that the *Smvkr2* gene was more actively transcribed than the *Smvkr1*gene (2.6 fold more) (Figure S2,A). This result is in accordance with the detection of *Smvkr2* transcripts preferentially found within the part of the ovary containing oogonia (Figure 1,B). Moreover, qPCR data indicated that both *Smvkr1* and *Smvkr2* transcripts were up-regulated strongly (13.5 fold and 5.4 fold respectively) in the ovaries of sexually-developed females as compared to the organs from virgin females issued from unisexual infections (Figure S2,B). This indicated the importance of SmVKR receptors during development and maturation of reproductive organs. The up-regulation of *Smvkr1* could be related to primary oocytes, which dominate within the posterior part of the ovary (Figure 1,B). Q-PCR analyses of isolated testes confirmed the presence of *Smvkr1* and *Smvkr2* transcripts in male reproductive organs and showed their up-regulation in testes from males issued from bisexual infections (data not shown).

### Effect of SmVKR gene suppression on female reproductive organs

**Figure 2:**
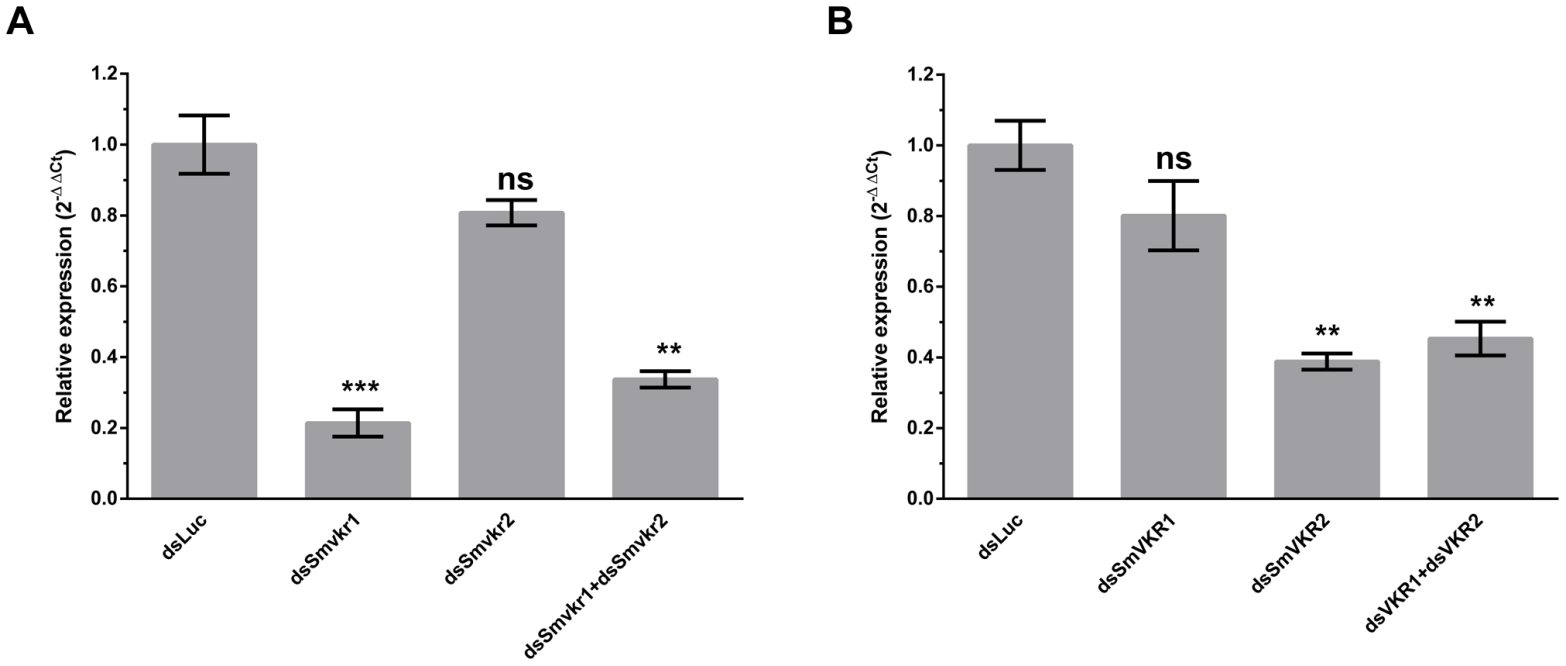
Efficiency of SmVKR1 and SmVKR2 knock-down by RNA interference in adult worm pairs. Worm couples were electroporated and incubated for 5 days either with dsSmvkr1 (20 µg) or dsSmvkr2 (20 µg) or with both of them (10 µg each) as described in Materials and Methods. Control worms were treated in the same conditions with irrelevant dsLuc RNA (20 µg). Levels of *Smvkr1* (A) and *Smvkr2* (B) transcripts were determined by quantitative RT-PCR in each worm sample. RNAi treatment with dsSmvkr1 or with dsSmvkr2 results in specific reduction of *Smvkr1* and *Smvkr2* transcripts respectively. Treatment with both dsSmvkr1 and dsSmvkr2 affects the expression of both *Smvkr1* and *Smvkr2* genes. For graphical representation, the ΔΔCt method was used to evaluate the relative expression of transcripts in interfered parasites compared to control dsLuc-treated parasites. Statistical analysis was performed using the Student's t-test and values are expressed as mean+/− SEM of three determinations (** p≤0.01, *** p≤0.001).

*Smvkr* gene expression was targeted for suppression by introducing via electroporation *Smvkr1* or *Smvkr2* dsRNA in couples of adult parasites *in vitro*. Figure 2 shows that a 5 day-treatment of worm couples with dsSmvkr1 led to a decrease of about 80% of the amount of *Smvkr1* transcripts in total parasites with a non-significative reduction of the level of *Smvkr2* transcripts, indicating a priority targeting of the *Smvkr1* gene by dsSmvkr1. Similar treatment with dsSmvkr2 gave comparable results, showing a reduction of more than 60% of *Smvkr2* transcripts but only a small and non-significative decrease of *Smvkr1* transcripts. Concomitant introduction of both dsSmvkr1 and dsSmvkr2 induced a simultaneous and significant reduction of both transcripts (70% and 55% for *Smvkr1* and *Smvkr2* respectively).

**Figure 3:**
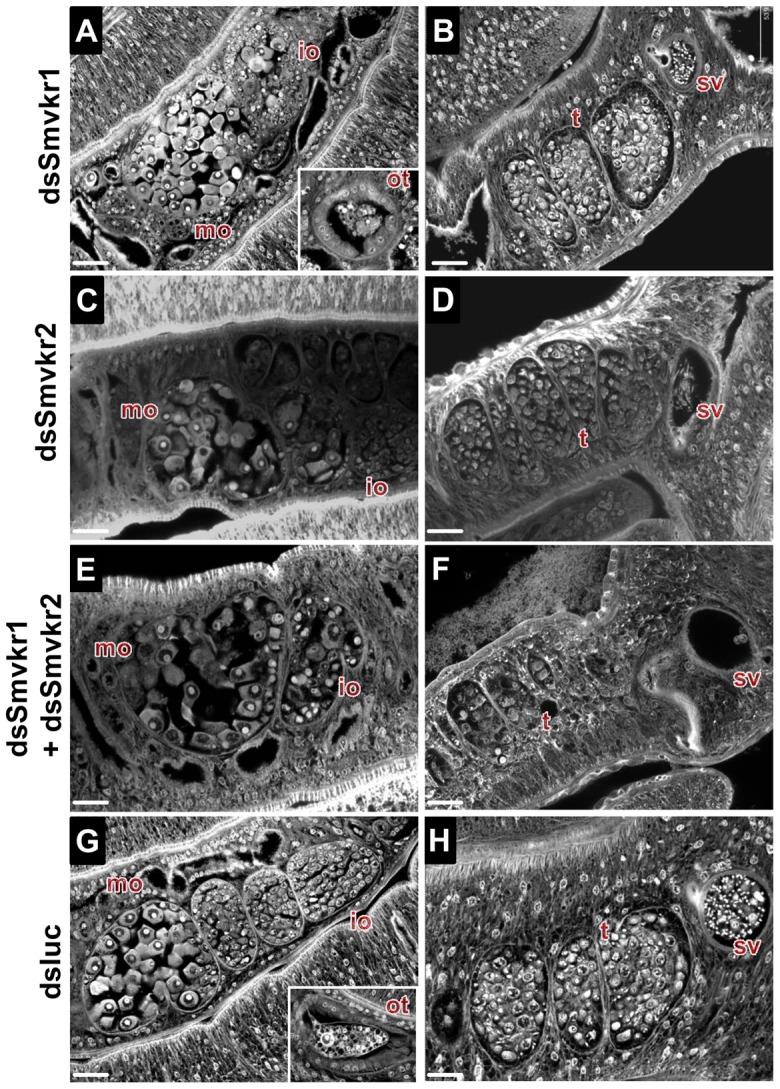
Morphological analysis of reproductive organs from worms treated with dsSmvkr1 or dsSmvkr2 RNA. CLSM images of whole-mount preparations of *S. mansoni* worm couples stained with carmine red. Worms were treated exactly as described in Fig. 2 with dsSmvkr1 (A, B), dsSmvkr2 (C, D), dsSmvkr1 and dsSmvkr2 (E, F) or control dsLuc (G, H) RNA. The morphology of female (left) and male (right) reproductive organs was analyzed. io: immature oocytes, mo: mature oocytes, ot: ootype, sv: sperm vesicle, t: testes. Scale bar: 20 µm.

The suppression of *Smvkr* gene expression did not result in any detectable changes in worm behavior and male-female pairing. To investigate whether RNAi-mediated silencing of *Smvkr* genes could have any impact on the morphology of parasite reproductive organs, we examined the worm couples after 5 days of dsRNA treatment by using CLSM (Figure 3). In male worms we did not observe any significant changes in the morphology of testicular lobes of both dsSmvkr1- and dsSmvkr2-treated parasites for which we noted the presence of sperm in seminal vesicle (Figure 3B,D). However, in double-targeted worms, we observed frequently a reduced diameter of the testicular lobes accompanied by a reduction of cell density in testes as well as empty seminal vesicles, compared to single dsSmvkr-treated worms or to control worms treated with dsLucRNA (Figure 3F). In female worms, drastic changes in the structure and size of the ovary appeared when parasites were treated with dsSmvkr1 or dsSmvkr2 or both of them, compared to control dsLucRNA (Figure 3A,C,E,F). Structure and content of the ovary, normally composed of differentially matured oocytes, i.e., small oogonia in the anterior part and primary oocytes within the posterior part, were drastically changed in *Smvkr*-suppressed parasites. A strong disorganization of the ovary containing predominantly big cells was observed following dsSmvkr1 or dsSmvkr2 treatment (Figure 3 A,C), and this phenotype was associated to a strong reduction of the ovary size in dsSmvkr2-treated females (Figure 3C). When both genes were targeted, we observed an addition of the two phenotypes (Figure 3E). The bottom insert (Figure 3,G) shows the presence of an egg formed by a fertilized oocyte and 30–40 vitellocytes in the ootype of dsLuc-treated control worms. Within the ootype of dsSmvkr1-targeted females, clusters of unorganized vitelline cells and the absence of a regular egg shell were identified indicating problems in egg formation (upper insert Figure 3,A). These results confirmed that SmVKR1 and SmVKR2 are important for gametogenesis, oogenesis and perhaps also for egg formation and thus are implicated in the reproductive functions of *S.mansoni*. Moreover, obtaining distinct phenotypes in dsSmvkr1 and dsSmvkr2-treated females could corroborate the different localization of the receptors in the ovary, and suggested possible different functions in this organ. The mode of activation and the signalling pathways potentially elicited by SmVKR1 and SmVKR2 respectively, were further studied.

### Activation of SmVKR1 and SmVKR2 by distinct ligands

**Figure 4:**
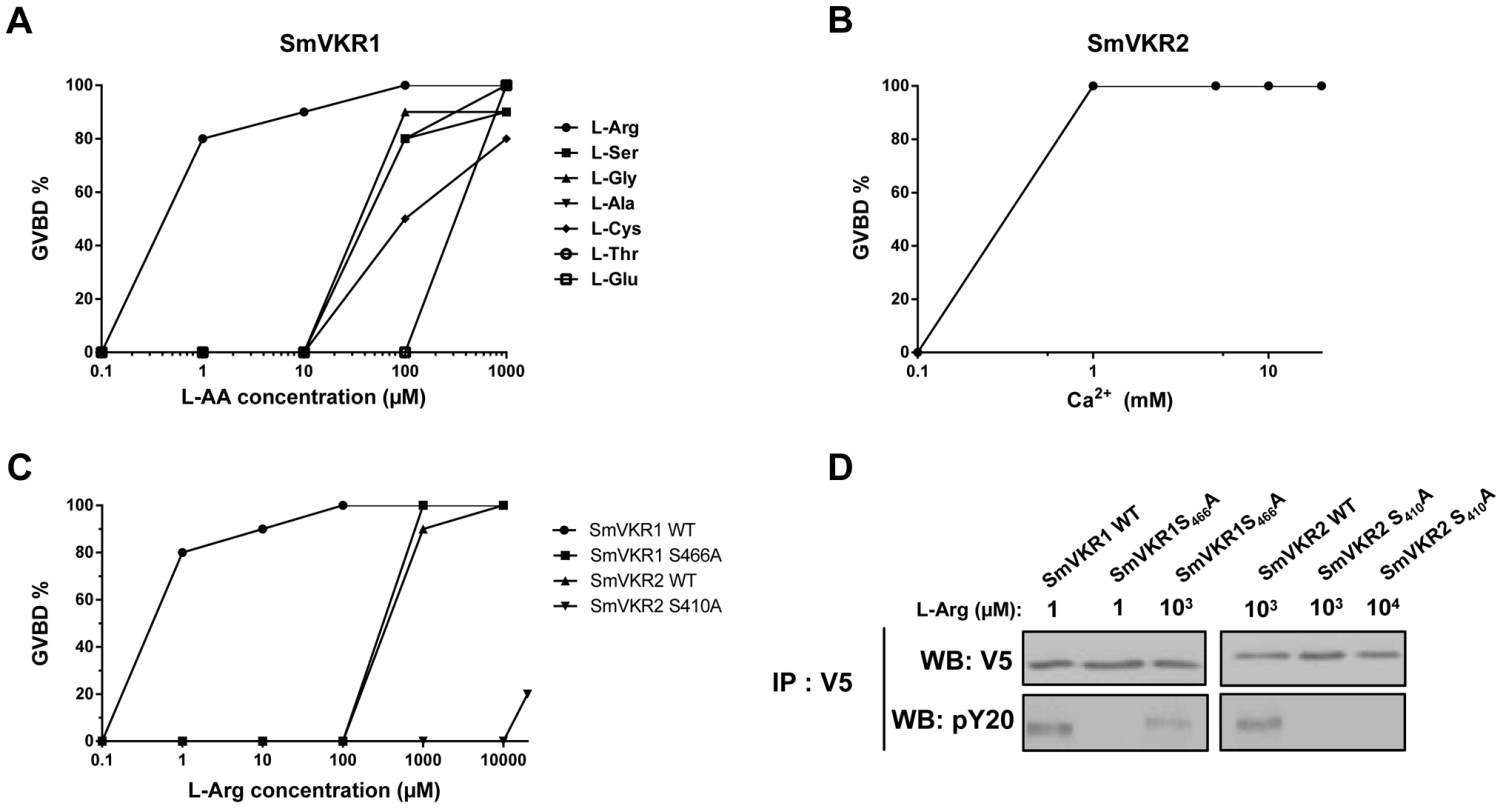
Ligand determination of SmVKR1 and SmVKR2. Importance of a conserved Ser residue of the VFT domain in amino-acid binding and activation of the receptors. A) Dose-effect of selected amino acids on the activation of SmVKR1 and potential to induce GVBD in *Xenopus* oocytes. L-Arg is the most potent activator of SmVKR1 and is active at 1 µM. B) Dose-effect of Ca^2+^ on the capacity of SmVKR2 to induce GVBD. C) Importance of Ser_466_ on the potential of L-Arg to activate SmVKR1. SmVKR1 mutated on its Ser_466_ residue present in the VFT domain requires 10^3^ fold higher amounts of L-Arg to be activated. The mutation of Ser_410_ in the VFT of SmVKR2 (which can be activated by 1 mM L-Arg in the absence of Ca^2+^) also abolishes its capacity to be activated by L-Arg, confirming the importance of this Ser residue highly conserved in the amino acid binding motif of VFT [28], [33]. All experiments have been repeated three times and the mean percentages of GVBD are indicated. D) Western blot analysis of membrane extracts of oocytes expressing wild-type and mutated SmVKR proteins confirming the expression of the receptors and indicating their level of phosphorylation (activation) following L-Arg binding. SmVKR1S_466_A is not recognized by anti-phosphotyrosine antibodies in the presence of L-Arg 1 µM and SmVKR2S_410_A is no more phosphorylated with L-Arg even added at 10 mM.

VKRs form a family of structurally conserved transmembrane receptors, composed of a TK intracellular domain and an extracellular VFT domain that serves for ligand binding and subsequent receptor activation. It was shown previously that VKR from the insect *Apis mellifera* was activated by amino acids, particularly by arginine [28] and the capacity of L-Arg to activate SmVKR1 was then demonstrated. Indeed, following receptor expression in *Xenopus* oocytes, binding of L-Arg induced SmVKR1 autophosphorylation and the activation of pathways leading to resumption of meiosis in the oocytes ([30], Figure 4). In former studies, we showed that meiosis resumption was dependent on kinase activation [17], [18], [30] leading to Germinal Vesicle BreakDown (GVBD), a process easily detectable by the formation of a white spot at the animal pole of the oocyte. According to this, we used this assay system to test the ability of all proteinogenic L-amino acids to behave as ligands and to activate SmVKR1 or SmVKR2 respectively. Results showed that seven L-amino acids (Arg, Ser, Gly, Ala, Cys, Thr and Glu) had the potential to activate SmVKR1 at a 1 mM concentration (Table S1) and also confirmed that L-Arg was the most potent agonist for SmVKR1 (Figure 4,A). When added at 1 µM in the oocyte incubation medium, L-Arg was able to induce GVBD in 80% of the SmVKR1-expressing oocytes. Surprisingly, expression of SmVKR2 in oocytes induced spontaneously their maturation in the usual ND96 medium, a minimally buffered saline solution complemented with CaCl_2_ (2.8 mM) and MgCl_2_ (1 mM). This observation suggested that bivalent ions could be responsible for the spontaneous activation of SmVKR2 and the use of salt-depleted media further indicated that Ca ions (at a 1 mM minimal concentration) were effectively responsible for the activation of SmVKR2 (Figure 4,B). To assess the specific action of Ca^2+^, we tested the effect of other bivalent ions (Ni^2+^, Fe^2+^, Mn^2+^ and Cu^2+^) on SmVKR2-expressing oocytes, but none of these ions were able to induce GVBD, even when they were used at a 10 mM dose in the Ca^2+^-depleted medium (data not shown). Additional experiments performed in ND96 medium without Ca^2+^ demonstrated that the activation of SmVKR1 by 1 µM L-Arg was not affected by the absence of Ca^2+^. They also showed that L-Arg itself was able to activate SmVKR2 but at a minimal concentration of 1 mM, thus 1000- fold higher than that required for SmVKR1 (Figure 4,C). Three other L- amino acids (Thr, Trp and Cys) also activated SmVKR2 at 1 mM (Table S1).

SmVKR1 and SmVKR2 receptors share similar VFT modules (48% residue identity). VFT modules constitute the binding pocket of various receptors activated by small molecules [27]. In most class C GPCRs, they are the binding sites for natural amino acids or derivatives and ligand binding depends on a consensus motif of 8 residues implied in the recognition of the α-amino acid group (i.e. primary amine and carboxylic acid) [33]. Particularly, the Ser_165_ residue which binds the COOH group of glutamate in the metabotropic glutamate receptor (mGluR1) and is the most conserved residue in class C GPCRs, is highly conserved in all VKRs [28], [29]. In order to confirm the implication of this Ser residue in amino acid binding to SmVKR1 and SmVKR2, we mutated the Ser_466_ and Ser_410_ of SmVKR1 and SmVKR2 respectively in Ala, and the mutated receptors were expressed in oocytes. GVBD assays were performed using L-Arg as a ligand. We found that a 1000-fold higher concentration of L-Arg (1 mM) was required to activate the mutated SmVKR1S_466_A and to induce GVBD. Similarly, SmVKR2S_410_A failed to respond to L-Arg (Figure 4,C). Western blot results confirmed that such differences in L-Arg sensivity were not due to a lower level of expression of the mutant receptors in oocytes. Wild type and mutated forms of SmVKR1 and SmVKR2 were detected with the same intensity in oocyte membrane extracts by anti-V5 antibodies. On the same blots, anti-phosphotyrosine (PY) antibodies revealed that phosphorylation of SmVKR1 and SmVKR2 perfectly correlates to their ability to induce GVBD in oocytes (Figure 4,D). These data confirmed the importance of the conserved Ser residue in amino acid binding.

Among all amino acids, Arg is uniquely containing a guanidino group. Therefore, we investigated the importance of this group in the affinity of L-Arg to SmVKR1 by testing a panel of Arg derivatives devoid either of the guanidino group (ornithine), or of the α-amino acid function (creatine, agmatine) as well as two Arg analogs (D-Arg, Canavanine). Results (not shown) indicated that all these compounds were inactive on SmVKR1 and that all of them had the potential to compete and to block totally the inducing effect of L-Arg when added with a 10-fold excess ratio. Such data are in favor of a specific activating effect of L-Arg on SmVKR1, which very likely implies a precise recognition of the amino groups of the Arg molecule together with specific constraints for a correct positioning of the ligand. Further studies of structure-activity relationships are needed to elucidate the nature of the residues involved in the interaction between SmVKR1 and L-Arg.

### SmVKRs function as homo- and heterodimers

**Figure 5:**
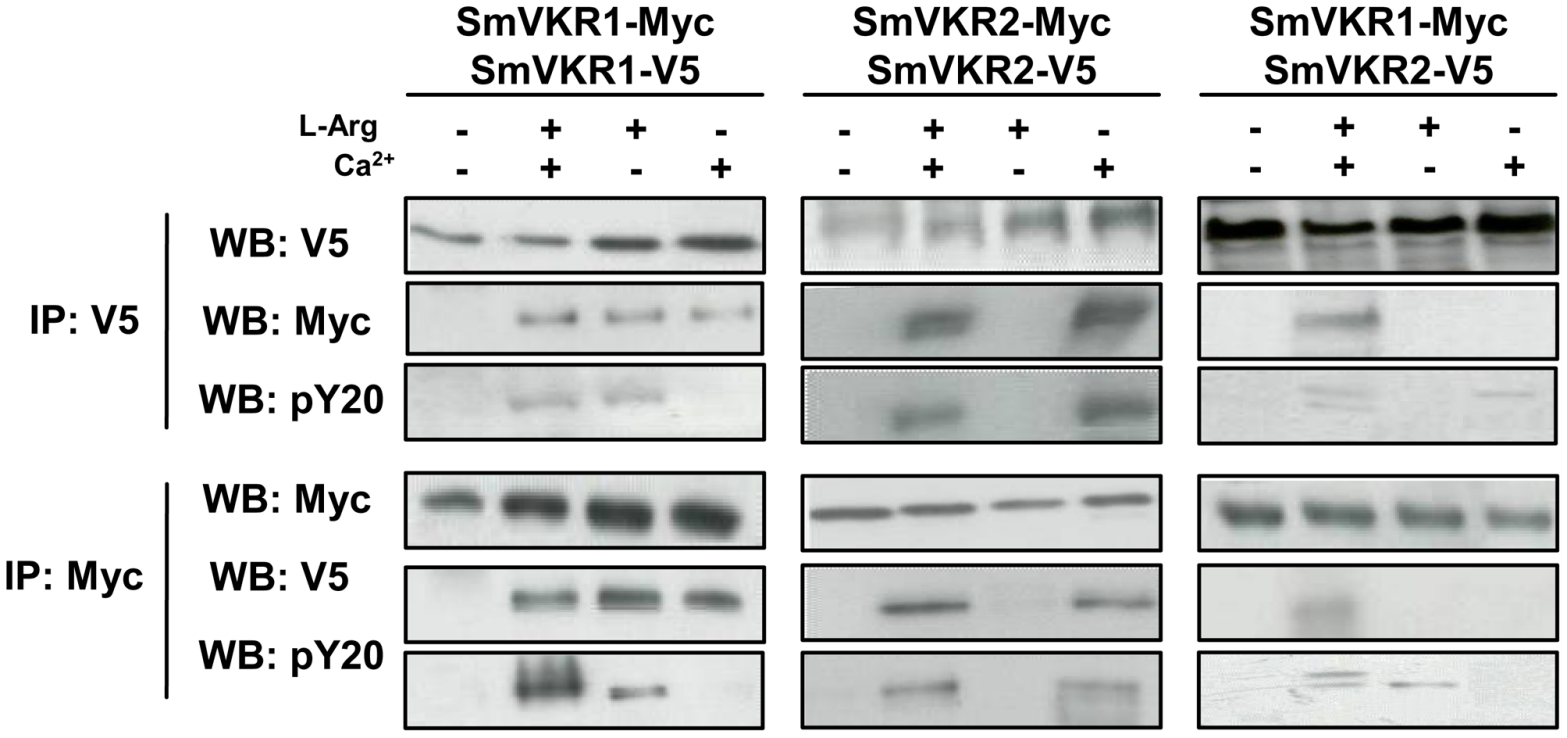
Homo- and hetero-dimerisation of SmVKR. Requirement of Ca^2+^ and/or L-Arg for receptor dimerisation and kinase activation. cRNAs encoding different tagged versions (Myc or V5) of SmVKR1 and SmVKR2 were injected in *Xenopus* oocytes. Oocytes were incubated for 5 h in ND96 medium with or without Ca^2+^ and L-Arg, as indicated. Membrane soluble extracts were immunoprecipitated (IP) by anti-V5 or anti-Myc antibodies and analysed by Western Blot (WB) with anti-V5, anti-Myc or anti-phosphotyrosine (pY20) antibodies. L-Arg and Ca^2+^ induce respectively the formation of active homodimers of SmVKR1 and SmVKR2. SmVKR1/SmVKR2 active heterodimers can be formed in the presence of L-Arg and Ca^2+^ (two bands of 170 kDa and 150 kDa corresponding respectively to SmVKR2 and SmVKR1 are labelled by anti-PY antibodies).

RTK activation is known to require homo- or heterodimerization of receptor molecules and similarly, it has been clearly demonstrated that the VFT modules of class C GPCRs also function as dimers [27]. A three dimensional model of the VKR of *A. mellifera* was previously built which suggested that a VFT dimer interface was present in VKR proteins similar to that in GPCRs. It was shown further that AmVKR proteins effectively form dimers at the cell surface [28]. In this work, we have investigated the capacity of SmVKR proteins to homo- or heterodimerize. Results in Figure 5 demonstrate that SmVKR1 and SmVKR2 are active as dimers when they are expressed in *Xenopus* oocyte. Using two versions of SmVKR1 differentially tagged with V5 or Myc epitopes, we showed by co-immunoprecipitation and Western blot analysis that SmVKR1 proteins can form homodimers at the oocyte membrane. The interaction between SmVKR1-V5 and SmVKR1-Myc molecules required the presence of Ca^2+^ or of L-Arg. However, as shown by labelling with anti-PY antibodies, SmVKR1 phosphorylation and thus kinase activation were dependent on the addition of L-Arg and could not be induced by Ca^2+^ alone. Similarly, analyses performed with SmVKR2 showed the effect of Ca^2+^ on receptor dimerization but also on its activation, both processes being independent on the addition of L-Arg (Figure 5). These data thus confirmed the previous demonstration that SmVKR1 and SmVKR2 were activated respectively by L-Arg and Ca^2+^. Even if in the developing ovary, the detection of SmVKR1 and SmVKR2 transcripts at different sites is not in favor of their possible interaction in this organ, we cannot exclude that in certain tissues (for example in testes where they are equivalently distributed in the whole organ) the two receptors can co-interact and co-activate at the cell membrane, similarly to many other RTKs. Following co-expression of SmVKR1 and SmVKR2 in *Xenopus* oocytes, we showed effectively that they were able to interact and that this interaction occurred only when both the ligands L-Arg and Ca^2+^ were added. In these conditions, two bands were revealed by anti-PY antibodies in the precipitated heterocomplexes, corresponding respectively to activated SmVKR1 (150 kDa) and SmVKR2 (170 kDa) (Figure 5). When only Ca^2+^ or only L-Arg was added, a single band was revealed by anti-PY antibodies in immune complexes precipitated either by anti-V5 or by anti-Myc antibodies. These bands likely represent activated homodimers of SmVKR2-V5 and SmVKR1-Myc, respectively.

### Identification of SmVKR interacting partners

The identification of potential SmVKR signalling partners was performed by yeast two-hybrid (Y2H) screening of a *S. mansoni* cDNA library [34], using the intracellular domains (ICD) of SmVKR1 or of SmVKR2 as baits. ICD mutants exhibiting constitutive kinase activity due to the introduction of a negatively charged glutamic residue next to the YY autophosphorylation site [30], [35] (SmVKR1ICD^YYRE^ and SmVKR2ICD^YYRE^) were used as baits in order to facilitate the capture of partners interacting with the receptors in their activated/phosphorylated state. About 400 clones positive for β-galactosidase expression were selected. Prey plasmids were isolated and their sequences determined. Among these, we could identify by BLASTn analyses 55 and 15 potential protein partners for SmVKR1 and SmVKR2 respectively, which were classified according to their putative functions (Table 1). Several proteins were selected by both SmVKR1 and SmVKR2, like actin and prefoldin involved in cytoskeleton functions and coatomer subunit with roles in membrane trafficking. The NAD-dependent deacetylase sirtuin-7 involved in chromatin remodelling was also shown to interact with both ICDs. Concerning proteins involved in phosphosignalling, we noted the selection of two important molecules Cbl and Shb, already known for their direct interaction with RTK. The E3 ubiquitin-protein ligase Cbl was only trapped by SmVKR2 and this fact might be corroborated by the prediction using UbPred program [36] of more ubiquitination sites in SmVKR2 than in SmVKR1. Secondly, an SH2 domain-containing adapter of the Shb protein family was demonstrated to bind specifically to SmVKR1 in its phosphorylated form and the role of the SH2 domain of SmShb in this interaction was recently demonstrated (results not shown). Two other proteins (XM_002575792.1 and XM_002574592.1) were selected with SmVKR1 but not with SmVKR2. Using phylogenetic analyses, the first one was shown to belong to the subfamily of protein phosphatase PP2C/PPM1G gamma subfamily (Figure S4) and the second one was shown to belong to the MEK7 subfamily of the MAPK kinase family (Figure S5). As protein phosphatases PP2C are known to be regulators of the activity of stress-induced MAPK pathway components [37] and as MEK7 proteins are responsible for JNK activation [38], it was interesting to further analyse the processes of signalling by these two receptors in order to potentially discriminate distinct pathways for each VKR.

**Table 1:** List of proteins identified by yeast-two-hybrid analyses using intracellular domains of SmVKR1 and SmVKR2 as baits.

Functional group	Identified proteins		Accession N°	Identified with		Overexpression in ovary	
				SmVKR1	SmVKR2	*S. mansoni*	*S. japonicum*
**Cytoskeleton arrangement**	Fibrillin 2		U54588.1	✗			
	Rho GTPase (SmRho1)	*	AY158212.1	✗		✓	✓
	Spectrin, Beta chain		XM_002579838.1	✗			✓
	Zyxin/trip6		XM_002575737.1	✗			
	Actin		XM_002580135.1	✗	✗	✓	✓
	Prefoldin subunit		XM_002578485.1	✗	✗	✓	✓
**Membrane trafficking**	Junctophilin-like protein		XM_002576132.1	✗			
	Synaptotagmin		XM_002576619.1	✗		✓	✓
	Coatomer gamma subunit		XM_002580203.1	✗	✗		
**Gene expression**	Expressed protein (similar to ARID2)		XM_002577690.1	✗			
	Expressed protein (Btz domain)		XM_002572591.1	✗			
	Ftz-F1 interacting protein		AY456264.1	✗			✓
	HRX histone methyltransferase		XM_002574332.1	✗			
	Notch		XM_002574857.1	✗			✓
	Nuclear autoantigenic sperm protein		XM_002577077.1	✗			✓
	Peter-pan related protein		XM_002580320.1	✗		✓	✓
	Plac8 homolog		XM_002573812.1	✗		✓	
	Pre-mRNA processing protein prp39-related		XM_002579875.1	✗		✓	✓
	TFIIA Large subunit		XM_002579091.1	✗		✓	✓
	tRNA d(2)-isopentenylpyrophosphatase		XM_002571087.1		✗		
	Cellular nucleic acid binding protein		XM_002581028.1	✗	✗	✓	
	Sirtuin 7 (Sirt7)	*	KC993857.1	✗	✗	✓	
**Phosphosignalling**	Mek7	*	XM_002574592.1	✗			✓
	PP2C	*	XM_002575792.1	✗		✓	✓
	Shb-like protein	*	JN864885.1	✗			
	Cbl		XM_002571919.1		✗	✓	
**Protein synthesis**	Elongation factor 1-alpha		Y08487.1	✗		✓	✓
	Methionyl aminopeptidase 2 (M24 family)		XM_002572527.1	✗		✓	
	Ribosomal protein S2		XM_002572774.1	✗			
	40S ribosomal protein S28, putative		XM_002573998.1	✗		✓	✓
	PDE-12 like protein	*	XM_002577997.1		✗	✓	
	60S ribosomal protein L26-like		DQ480541.1		✗		✓
	Elongation factor Tu GTP binding domain containing 1 isoform 6-related		XM_002571419.1	✗	✗		
**Diverse enzymes**	ATP-dependent transporter		XM_002576209.1	✗			✓
	Autophagy-related protein 101 like		XM_002577493.1	✗			
	Carboxypeptidase		XM_002581470.1	✗			✓
	Cathepsin B		M21309.1	✗		✓	✓
	Chitobiosyldiphosphodolichol alpha-mannosyltransferase		XM_002571918.1	✗			
	GAPDH		XM_002576948.1	✗			✓
	Glutathione peroxidase		M86510.1	✗			✓
	Microsomal glutathione s-transferase		XM_002576747.1	✗			✓
	NADH-cytochrome B5 reductase		XM_002576573.1	✗			✓
	NADH-ubiquinone oxidoreductase 24 kD subunit		XM_002578142.1	✗		✓	✓
	Ornithine aminotransferase		EU042598.1	✗			✓
	Peptidylprolylisomerase		XM_002577985.1	✗			
	Subfamily T1A non-peptidase homologue (T01 family)		XM_002574652.1	✗		✓	
	Thimet oligopeptidase (M03 family)		XM_002574355.1	✗			✓
	Zinc metalloproteinase		XM_002573446.1	✗			
	26S protease regulatory subunit S10b		XM_002573132.1	✗			
	Endoglycosylceramidase		XP_002577487.1		✗		

Proteins were classified in functional groups and Genbank accession numbers are indicated. Ticks indicate sequences already shown to be overexpressed in ovary by transcriptomic analyses of *S. mansoni* [69] and *S. japonicum* [70]. Asterisks indicate the proteins for which the interaction of full length sequences with VKR has been confirmed in yeast.

### Studies of SmVKR1 and SmVKR2 signalling pathways

**Figure 6:**
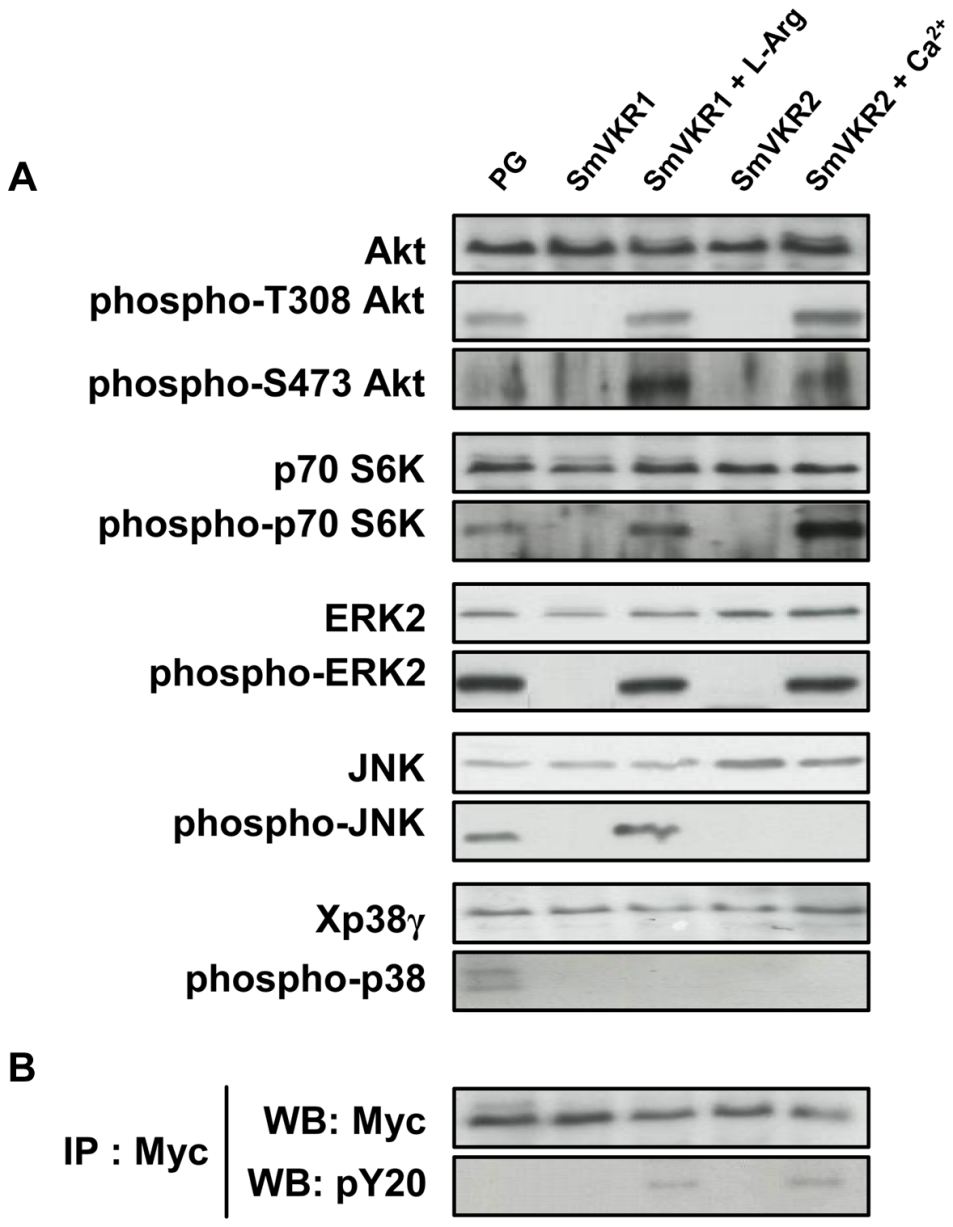
Analysis of signaling pathways triggered by SmVKR activation in *Xenopus* oocytes. SmVKR1-Myc and SmVKR2-Myc proteins were expressed in *Xenopus* oocytes for 5 h in ND96 incubation medium with or without their respective ligands (L-Arg 1 µM and Ca^2+^ 1 mM). **A**-Oocyte lysates were analyzed by Western blot to detect the phosphorylation state of Akt, S6K, ERK2, JNK and p38γ following SmVKR1 and SmVKR2 activation by L-Arg and Ca^2+^ respectively. As a control, SmVKR1-expressing oocytes were stimulated by progesterone (PG), the natural hormonal stimulus for oocyte maturation. Results show that both SmVKR1 and SmVKR2 activate Akt and Erk2 pathways. S6K can be phosphorylated by both receptors, but more importantly by SmVKR2. Only SmVKR1 can trigger JNK activation. The p38γ pathway, is activated in control PG-stimulated oocytes, but not under activation of SmVKR1 or SmVKR2. **B**-Immunoprecipitation of oocyte extracts by anti-Myc antibodies allowed us to confirm the expression of receptors (WB anti-Myc) and their activation/phosphorylation (WB anti-pY20) in the presence of their respective ligands.

From the indications that SmVKR1 and SmVKR2 i) were expressed differentially in the tissues of *S. mansoni*, ii) were activated by distinct ligands, and iii) were able to interact with different partners in the yeast expression system, we explored the possibility that these receptors could mediate distinct signalling pathways in the parasite. The *Xenopus* oocyte is a convenient model system to study signalling pathways initiated by extracellular inducers. Thus, we analyzed in oocytes expressing SmVKR1 and SmVKR2, the effect of L-Arg or of Ca^2+^ on the phosphorylation of key cellular proteins already known to be involved in RTK signalling, and particularly those belonging to PI3K/Akt/mTOR and MAPK pathways. In controls, we treated oocytes with progesterone (PG), a natural stimulus known to induce activation of these pathways in stage VI *Xenopus* oocytes [39]. Analyses of phosphoproteins in oocyte extracts were performed 5 hours after the addition of L-Arg and Ca^2+^ ligands and prior to oocyte maturation, in order to anticipate the phosphorylation loops which will set up following the activation of MPF (Maturation Promoting Factor). Results in Figure 6 show that activation and phosphorylation of both SmVKR1 and SmVKR2 elicit in the oocyte the activation of the PI3K pathway resulting in the phosphorylation of the kinase Akt/PKB by its activating kinases PDK (phosphoinositide-dependent kinase) and mTORC2 (mammalian target of rapamycin complex 2) respectively on Thr_308_ and Ser_473_ residues. Akt is known to activate mTOR leading to the activation of p70S6K, a mitogen activated kinase responsible for the phosphorylation of the S6 protein of the 40S ribosomal subunit, and involved in the translational control of ribosomal proteins and elongation factors [40]. The phosphorylation of p70S6K on Thr_389_, a residue critical for its kinase activity, was detected in the oocytes following activation of SmVKR1 and SmVKR2 by their respective ligands. Such profiles were similar to those obtained in PG-stimulated oocytes, confirming the activation of the PI3K/Akt/S6K pathway by SmVKR1 and SmVKR2. No activation of the PI3K pathway was observed in the absence of ligand in SmVKR-expressing oocytes.

Activated RTKs constitute platforms for the recognition and recruitment of adaptor proteins with SH2 domains linking extracellular signals for RTK activation to downstream signal transduction pathways. Among these, the MAP kinase signalling cascade (Ras-Raf-MEK-ERK) plays a pivotal role and is essential for a variety of processes such as growth, differentiation, proliferation, survival and apoptosis in all eukaryotes. Using anti-phospho ERK2 antibodies, we showed that both SmVKR1 and SmVKR2 can activate downstream the canonical ERK/MAPK cascade.

JNK/MAPK pathway activation is also a classical component of RTK signalling [41], [42]. Our results show that SmVKR1 when activated by L-Arg induces the phosphorylation of JNK, exactly as does the activation of *Xenopus* receptors by PG or by insulin in control oocytes (see Figure 6 A and S3). However, JNK phosphorylation is not detected in the case of SmVKR2 activated by Ca^2+^ in spite of the potential of oocytes to undergo GVBD, and this is in agreement with the fact that JNK plays a limited role in cell-cycle progression [42]. The activation of JNK by SmVKR1 corroborated the identification of JNK pathway actors, like MEK7 and PP2C, as potential interacting partners of SmVKR1 but not of SmVKR2 (Table 1), providing evidence that each VKR can elicit common but also distinct pathways. Additional data (Figure S3) also indicated that the autophosphorylation of JNK was inhibited by its specific inhibitor SP600125 [43] but not by the addition of purvanalol, the inhibitor of the cyclin-dependent kinase CDK1 that is responsible for MPF activation and meiosis resumption in the oocyte. Therefore, JNK activation, which is elicited by SmVKR1 but not by SmVKR2, follows a pathway parallel to meiotic processes and this confirms the potential of SmVKR1 in specific functions.

In higher eukaryotes, activation of p38 MAPK usually correlates with cell cycle arrest. However, in *Xenopus* oocytes, p38γ/SAPK3 activation has been shown to be important for the meiotic G2/M progression [44]. Following activation by its upstream kinase MKK6, it phosphorylates and activates Cdc25C, the phosphatase directly responsible for MPF activity. Active mutants of MKK6 accelerate PG-induced maturation in oocytes whereas kinase-dead mutants of MKK6 or p38γ inhibit this meiotic progression [45]. Results in Figure 6 A confirm this activation of p38 MAPK in PG-stimulated oocytes but they clearly indicate that SmVKR1 and SmVKR2 kinases are not able to induce this pathway.

## Discussion

VKR is an uncommon RTK that bears a VFT ligand-binding domain not found in any other RTK [26]. Since its first discovery ten years ago in *S. mansoni*, VKR has been found in a large variety of organisms. It is preferentially expressed in larval stages and in gonads of several organisms, suggesting its role in development and reproduction [28]. This work is centered on the functions of VKR in *S. mansoni*, and specially on the demonstration of its role in reproduction. We performed a comparative analysis of the two members SmVKR1 and SmVKR2 expressed in *S. mansoni*, studying their respective tissue distribution, the morphological and physiological impact of their knock-down expression in parasites, molecular aspects of receptor activation and cellular signalling pathways.

Firstly, we showed by *in situ* detection of transcripts that SmVKRs were expressed in parasite reproductive organs, more intensively in female ovaries than in testes, in which only a diffuse labelling was obtained. These data were in agreement with the previous observation that VKRs were massively expressed in female gonads of insects and sea urchin, and thus corroborated the hypothesis of a role of VKR in reproductive processes. Additionally, the hints towards distinct localizations of SmVKR1 and SmVKR2 inside of the schistosome ovary suggested that each receptor could potentially initiate different processes in gametogenesis and egg production. Within the vitellarium, a tissue devoted to intense mitotic activities and in which different PKs known to be involved in mitosis are largely expressed (SmPlk1and SmSak polo-like kinases [17], [18], SmTK3 Src kinase [13] and TGFβ receptor SmTR1 [20]), *Smvkr* transcripts were not detected. This indicated that SmVKRs could play specific functions in gonads, particularly in gamete differentiation.

Recent data have shown that the well-known IR inhibitor, tyrphostin AG1024, is able to inhibit with a similar efficiency the kinase activities of SmIR1 and SmIR2 but also those of SmVKR1 and SmVKR2, hence confirming the IR-like TK properties of the four receptors [24]. Following AG1024 treatment of female parasites, a remarkable reduction of the size of the ovary was observed with a dominance of primary oocytes within the whole ovary. Additionally, the drug had a visible impact on the formation of the egg. In male schistosomes, AG1024 also affected spermatogenesis and differentiation of sperm [24]. These results already indicated that AG1024–sensitive IR-like receptor signalling played essential functions in gametogenesis. In the present study, we used RNA interference to target specifically SmVKR1 or/and SmVKR2 and to demonstrate that SmVKR were actually important actors for gamete production, oocyte maturation and egg formation.

**Figure 7:**
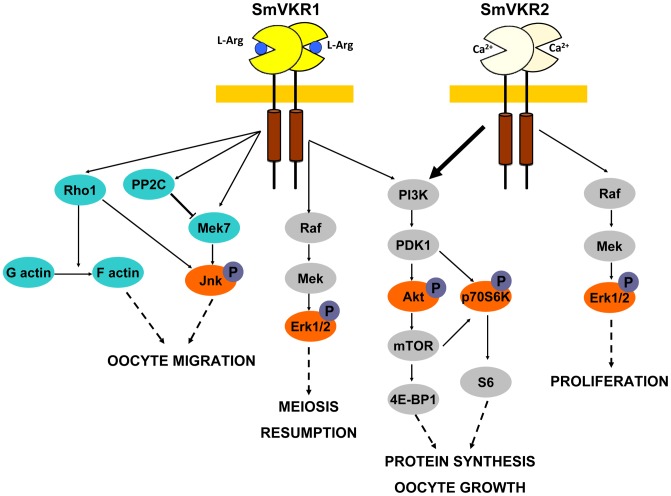
Summary of the major signalling pathways triggered by the activation of SmVKR and potentially regulating fate and differentiation of oocytes in female schistosome organs. Proteins identified by Y2-H screening (blue) and kinases detected for their activation in *Xenopus* oocytes (orange) are indicated. Proved and direct interactions between proteins are indicated by full arrows.

In male worms, targeting of SmVKR1 or SmVKR2 was not sufficient to clearly affect the production of sperm but when both SmVKRs were knocked down simultaneously, a modification of the cell density in testes was noticed in a large proportion of worms, which contained no more sperm in their seminal vesicles. From this result, it can be hypothesized that SmVKR1 and SmVKR2 may be co-expressed in male germinal cells, exerting redundant cellular functions in spermatocytes. However, we cannot fully exclude that subtle differences exist which are not detectable by this approach since the affected structures are minor compared to their clearly visible counterparts within the ovary. In female worms, the consequences of the suppression of SmVKR are spectacular, with important morphological changes in the ovary, following treatment with dsSmvkr1 or dsSmvkr2 or both. In all conditions, we observed a strong disorganization of the ovary which was dominated by the presence of primary oocytes. Additionally, in the case of dsSmvkr2 a reduction of the ovary size was found and in Smvkr1-suppressed worms an abortion of egg formation. These data confirm the essential roles of SmVKRs for female gametogenesis and egg production. Furthermore, the differences between the phenotypes observed in dsSmvkr1 or dsSmvkr2-treated parasites suggest that SmVKR1 and SmVKR2, could exert different functions at different steps of oocyte maturation (Figure 7).

Structural and functional differences between the two members of the VKR family in *S. mansoni* have been highlighted. Both SmVKR1 and SmVKR2 were shown to function as homodimers, as other insect VKRs [28] and we have shown that receptor dimerisation occurs for both SmVKR in the presence of Ca^2+^. The bivalent cation is sufficient to obtain dimerisation and activation of the kinase receptor in the case of SmVKR2 but not in the case of SmVKR1, which required to be activated that an amino acid, preferentially L-Arg, binds to its VFT domain. This property was found as common to all the insect VKRs studied ([28], not published). Thus, Ca^2+^ appears to be the main activator (and/or ligand) of SmVKR2 whereas L-Arg (or other amino acids with a lower efficiency), represents the ligand of SmVKR1. An important aspect of these studies concerns the difference in L-Arg-dependence for each SmVKR that is probably related to their different distribution inside the ovary and thus to the availability of ligands for oocytes along their maturation in reproductive organs. It is tempting to speculate that functional activity of SmVKR1, which is mainly present in mature, primary oocytes ready to be transported to the oviduct to be fertilized by sperm in the receptaculum seminis, is regulated by L-Arg since this amino-acid is largely known to be a major sperm constituent in many organisms [45], [46]. Considering as unlikely the formation of SmVKR1/SmVKR2 heterocomplexes in the ovary, their formation may be possible in the male gonad. Here, heterodimers could be active owing to the presence of L-Arg and Ca^2+^, which are both required to form active heterodimers, as shown in this study.

Following the demonstration by RNAi studies of the importance of SmVKR1 in schistosome reproduction, it was interesting to investigate the use, as an alternative to TK inhibitors, of antagonist ligands to block SmVKR1 activity. In this context, the fact that DmXR, a *Drosophila* GPCR with a VFT similar to that of all VKRs, could bind and be activated by the L-Arg analog, L-Canavanine [47], prompted us to check for the potential of various Arg derivatives, including L-Cana, to activate SmVKR1. Results indicated that none of the Arg derivatives tested was able to activate SmVKR1 and more importantly that all of them blocked in a competitive manner the effect of L-Arg on SmVKR1 activation. Preliminary experiments in which worms were incubated in the presence of L-Cana indicated that this L-Arg antagonist exerts a deleterious effect on reproductive organs comparable to that obtained with dsSmvkr1 treatment by RNAi (unpublished). This reinforces the idea that ligand mimics/antagonists could be, together with TK enzyme inhibitors (like tyrphostins) [24], efficient drugs to inhibit SmVKR activity and control reproduction in schistosomes. Of course, a better knowledge of the VFT structure of SmVKR1 is still required for the design of such antagonists and further SAR studies are currently in progress to identify the residues implied in L-Arg specific binding.

Not much is known about VKR signalling in schistosomes except that there is recent evidence for a cooperation of SmVKR1 with a kinase complex consisting of –in part- unusual kinases [16]. To enlarge the knowledge about binding partners of SmVKR1 but also SmVKR2, a cDNA library screening was performed using the Y2H protocol and intracellular parts of SmVKR1 and SmVKR2 with constitutive kinase activity in order to capture proteins interacting with the active receptors, and particularly those recognizing phosphotyrosines and containing SH2 or PTB domains. A large number of potential partners for SmVKR1 and SmVKR2 were trapped and they were classified in five groups according to their putative functions. Proteins involved in cytoskeleton reorganization, in vesicular trafficking, kinase signalling, gene expression or protein synthesis were captured by both SmVKR proteins, and they were more numerous in the case of SmVKR1. Several of these interacting partners possess known functions in reproduction. Among the specific SmVKR1 partners, we found SmRho1 (the RhoA GTPase already shown to be expressed in schistosome reproductive organs [34]), the kinase Mek7 and a phosphatase PP2C, three proteins involved in the JNK activation pathway [37], [38], [48]. Several evidences have pointed out diverse functions of the JNK pathway in germline homeostasis, meiosis progression and spindle assembly [49]–[51]. Additionally, a PP2C isoform (PPM1A) is synthesized during oocyte maturation in mammals [52] and the RhoA GTPase is involved in ovulation in *Caenorhabditis elegans* [53]. Another interacting partner of SmVKR1 is the transmembrane protein Notch, which is known to be involved in germline proliferation and meiosis progression [54]–[57]. Distinct SH2-containing adaptor proteins interacted with SmVKR1 and SmVKR2, these were SmShb and SmCbl, respectively. Interestingly, Shb was described to regulate oocyte meiosis I progression in mice and to be involved in ERK and RSK pathways [58]. Cbl is a E3 Ubiquitin-ligase expressed in *Drosophila* oocytes and responsible for dorso-ventral patterning of the ovary and germline proliferation [59], [60]. Finally, the SmVKR1 interacting partner Zyxin is a cytoskeletal protein that interacts with germline RNA helicases GLH in P granules in *C.elegans* [61].

From these data showing for each SmVKR different expression, ligand-activation and partner interactions, it seemed that SmVKR1 and SmVKR2 could elicit different cellular signalling pathways. In *Xenopus* oocytes, ligand-activated RTKs, and particularly insulin receptor, activate the Erk MAPK as well the PI3K/Akt/mTOR pathways. Using this cellular model, we observed that similarly to the insulin receptor, ligand-activated SmVKR1 and SmVKR2 both induce the phosphorylation of Erk1/2, Akt and p70S6K, indicating a potential role of SmVKRs in protein synthesis and cellular growth. Hyperphosphorylation of p70S6K in the case of SmVKR2 could be related to the predominant function in growth of SmVKR2 (Figure 7). Concerning the activation of the two other MAPK pathways, p38 and JNK, no phosphorylation of p38γ/SAPK3 was observed for SmVKR1 and SmVKR2 and JNK was only phosphorylated in SmVKR1-expressing oocytes, corroborating the results of Y2H screening and the finding that SmVKR1 interacted with Rho1, Mek7 and PP2C.

In conclusion, all these data give evidence of the roles of SmVKR in schistosome reproduction. RNAi results, together with previous results obtained with the use of TK inhibitors [24] confirm the functions of SmVKR in gametogenesis, and particularly in oogenesis and egg formation and the importance to consider these RTKs as potential targets against schistosomes in novel anti-fecundity therapies. Each receptor could be susceptible to act at different steps of oocyte maturation (Figure 7). SmVKR1, which is more expressed in mature ovocytes, would activate JNK, a pathway which is known to be involved in cell migration and meiotic progression. By this way, SmVKR1 might be responsible for meiosis resumption and/or ovocyte migration under activation by a male stimulus, L-Arg present in seminal fluid. SmVKR2 present in immature and dividing cells might be implied in priority in proliferation and growth of oocytes, thus explaining its preferential activation of the Akt/mTORC2/p70S6K pathway.

As a conclusion, we propose that VKR receptors might represent privileged targets to combat schistosomes, not only because of their role in parasite reproduction and thus their impact on pathogenesis and parasite transmission, but also because they have the main advantage over other parasite molecules to be absent from the host kinase panel. Moreover, as VKRs are also expressed in the gonads of disease-transmitting insects (like *Anopheles* vector of malaria or *Aedes* vector of filaria and viruses) [28], [29], and are probably involved in similar physiological processes of reproduction in these organisms, VKRs might also represent interesting drug targets for novel strategies to control other vector-borne infectious diseases besides schistosomiasis.

## Materials and Methods

### Ethics statement

All experiments involving hamsters within this study have been performed in accordance with the European Convention for the Protection of Vertebrate Animals used for Experimental and other Scientific Purposes (ETS No 123; revised Appendix A) and have been approved by the committee for ethics in animal experimentation of the region Nord Pas de Calais France (authorisation No. AF/2009) in the local animal house of the Pasteur Institute of Lille (Agreement No. A59-35009).

### Parasite material

A Puerto-Rican strain of *S. mansoni* was maintained by passage through albino *Biomphalaria glabrata* snails and *Mesocricetus auratus* golden hamsters. Adult schistosomes were collected by portal perfusion from infected hamsters at 42–45 days p.i, then maintained in complete M199 medium (supplemented with 10% SVF, 10 mM HEPES pH 7.4, 60 µg/ml Rifampicin and 50 U penicillin/streptomycin) at 37°C under a 5% CO2 atmosphere. Unisexual worm populations were generated by monomiracidial snail infection as described elsewhere [62].

### *In situ* hybridization

Adult worm pairs were fixed in Bouin's solution (picric acid/acetic acid/formaldehyde; 15/1/5) and embedded in paraplast (Paraplast plus, Sigma). Sections (5 µm) were incubated in xylol to remove paraplast. Following rehydration, slides were treated with proteinase K (1 µg/ml) and sections dehydrated. For hybridization, *in vitro* transcripts (corresponding respectively to nt 4057–4379 and 2617–3027 sequences of SmVKR1 (Genbank Acc N° AF101194) and SmVKR2 (Genbank Acc N° GU270860)) were synthesized and labeled with digoxigenin following the protocol of the manufacturer (Roche). The correct size of labeled sense and antisense transcripts was controlled by gel electrophoresis and the quality of RNA probes was checked by blotting and detection of digoxigenin using alkaline phosphatase-conjugated anti-digoxigenin antibodies, naphtol-AS-phosphatase, and Fast Red TR (Sigma). *In situ* hybridization was performed at 57°C for 16 h as previously described [34]. Sections were washed in 0,5×SSC (75 mM NaCl, 7,5 mM sodium citrate, pH 7) and detection of alkaline phosphatase on slides was achieved as described above.

### Quantification of SmVKR1 and SmVKR2 transcripts in ovaries isolated from unisexual or bisexual worms

Ovaries were isolated from female worms issued either from unisexual or bisexual schistosome infections according to the procedure described by Hahnel *et al* [31], and total RNA was purified from the gonad tissues using the PepGOLD TriFast reagent (Peplab) according to the manufacturer's protocol. For this 50 to 250 ovaries were incubated in 500 ml TriFast-solution frozen in liquid nitrogen and thawed on ice three times to support tissue disruption. Precipitation of total RNA in 2-propanol was driven by adding of 35 µg glycogen (RNase-free PeqGOLD glycogen, Peqlab). RNA quality and quantity were checked by electropherogram analysis employing the BioAnalyzer 2100 (Agilent Technologies).

Synthesis of cDNA was performed using the QuantiTect Reverse Transcription Kit (Qiagen) containing a genomic DNA wipe-out step and 200 ng of total RNA per reaction. The obtained cDNA was diluted 1∶20 and used in subsequent qPCR analyses. The detection of synthesized DNA double strands was based on the incorporation of SYBRGreen using PerfeCTa SYBR Green Super Mix (Quanta). All qPCR experiments were performed with a Rotor Gene Q Cycler (Qiagen) under the following conditions: 95°C for 3 min followed by 45 cycles of 95°C for 10 sec, 60°C for 15 sec and 72°C for 20 sec. SmVKR1qPCRF/SmVKR1qPCRR and SmVKR2qPCRF2/SmVKR2qPCRR2 primer couples (Table S2) were used to amplify SmVKR1 and SmVKR2 transcripts, respectively. All primers were designed to have melting points at 60°C and were commercially synthesized (Biolegio, Netherlands). To distinguish between specific amplification products and unspecific primer dimers following each qPCR analysis, a melting point analysis was done. Amplification reactions occurred in technical triplicates, and analyses were performed using a relative quantification against the reference gene actin (Smp_161930) with the ΔΔCt method [63].

### Microscopic examination of parasites

Worms fixed in AFA (ethanol 95%, formalin 3% and glacial acetic acid 2%) were stained for 30 min in 2,5% Hydrochloric Carmin red (Certistain®, Merck), and then destained in acidic 70% ethanol. Following dehydration in 70%, 90% and 100% ethanol, 5 min each, worms were preserved as whole-mounts in Canada balsam (Merck) on glass slides [11], [32], [64]. Confocal Laser Scanning Microscopy (CLSM) images were taken using a Leica LSM710 microscope with a 488 nm He/Ne laser and a 470 nm long-pass-filter under reflection mode.

### RNAi experiments

SmVKR1 (640 bp) or SmVKR2 (410 bp) cDNA fragments were generated as dsRNA templates by PCR amplification using gene specific primers containing T7 promoter sequences (see Table S2). A luciferase dsRNA template of similar size was generated using the pGL3-basic plasmid (Promega) as template. dsRNA was prepared and purified using the Megascript RNAi kit (Ambion) according to the manufacturer's instructions, and quantified spectrophotometrically (NanoVue PlusTM, GE Healthcare). 20 µg of dsRNA were delivered to 8 worm couples in 100 µL M199 medium by electroporation using the square-wave protocol already described (125 V, 20 ms impulse at room temperature in a 4 mm cuvette) [65]. Worms were then cultured in complete M199 medium. After 5 days, microscopic examination was performed by CLSM on 4 worm couples, as described above. Gene knock-down was monitored in the remaining worms by quantitative RT-PCR. RNA was extracted using the Dynabeads® mRNA DIRECT™ Micro Kit following manufacturer's instructions (Invitrogen). Reverse transcription was performed for 1 h at 55°C using the ThermoScript™ RT-PCR System for First-Strand cDNA Synthesis (Invitrogen) and cDNA was used as template for PCR amplification using KAPA SYBR® FAST Universal 2× qPCR Master Mix kit (Clontech) and the ABI PRISM 7300 detection system (Applied Biosystems, Foster City, CA, USA). SmVKR1qPCRF/SmVKR1qPCRR and SmVKR2qPCRF/SmVKR2qPCRR primer couples (Table S2) were used to amplify respectively SmVKR1 and SmVKR2 transcripts in triplicate assays. Tubulin gene (GenBank Acc N°M80214) was used as internal control. For graphical representation, the delta-delta Ct (ΔΔCt) method was applied [63] to compare interfered worms to control dsLuc-treated parasites. The statistical significance of the levels of SmVKR1 and SmVKR2 transcript knock-down was evaluated using Student's t-test in the GraphPad Prism programme (GraphPad Software Inc.).

### Site-directed mutagenesis

Full-length SmVKR1 and SmVKR2 sequences inserted in frame into the pcDNA3.1-V5/His expression vector (Invitrogen) [30] were mutated in their respective VFT domain by replacement of Ser_466_ and Ser_410_ residues into Ala using the QuickChange Site-Directed Mutagenesis Kit (Stratagene), and the SmVKR1S_466_AF and SmVKR2S_410_AF primers (Table S2) and their respective reverse complement sequences.

### Protein expression in *Xenopus* oocytes

cRNA encoding SmVKR proteins was synthesised *in vitro* using the T7 mMessage mMachine Kit (Ambion, USA) and PmeI-linearised SmVKR-pcDNA plasmids as templates, and injected in stage VI *Xenopus laevis* oocytes according to the procedure previously described [66]. Each oocyte was injected with 60 nl (60 ng) of cRNA in the equatorial region and incubated at 19°C in ND96 medium (96 mM NaCl, 2 mM KCl, 1 mM MgCl_2_, 1,8 mM CaCl_2_, 5 mM Hepes pH 7.4 supplemented with 50 µg.ml^−1^ Streptomycin/Penicillin, 225 µg.ml^−1^ sodium pyruvate, 30 µg.ml-1 trypsin inhibitor). Kinase activation was obtained by adding external ligands (amino-acids, Ca) in the incubation medium and Germinal vesicle breakdown (GVBD) was detected by the appearance of a white spot at the centre of the animal pole after 15 h.

### Immunoprecipitation and western blot analyses

Expression of SmVKR proteins in oocytes was confirmed by immunoprecipitation of membrane extracts according to the procedure described previously [66]. Following 24 h of expression, oocytes were lysed in buffer A (50 mM Hepes pH 7.4, 500 mM NaCl, 0.05% SDS, 5 mM MgCl2, 1 mg ml^−1^ bovine serum albumin, 10 µg ml^−1^ leupeptin, 10 10 µg ml^−1^ aprotinin, 10 µg ml^−1^ soybean trypsin inhibitor, 10 µg ml^−1^ benzamidine, 1 mM PMSF, 1 mM sodium vanadate) and centrifuged at 4°C for 15 min at 10,000 g. Membrane pellets were resuspended and incubated for 15 min at 4°C in buffer A containing 1% Triton X-100 and then centrifuged under the same conditions. Supernatants were incubated with anti-V5 or anti-Myc antibodies (1∶100; Invitrogen) overnight at 4°C. Protein A-Sepharose beads (5 mg; Amersham Biosciences) were added for 1 h at 4°C. Beads were washed three times and resuspended in Laemmli sample buffer. Eluted immune complexes were subjected to a 7.5% SDS–PAGE, then analyzed by Western blotting using anti-V5 (1∶50,000), anti-Myc (1∶50,000) or PY20 (1∶10,000; anti-phosphotyrosine, BD Biosciences) antibodies and the advanced ECL detection system (Amersham Biosciences).

Oocyte proteins phosphorylated during the activation of signalling cascades by SmVKR, were detected by Western blot analyses of oocyte lysates [67]. Oocytes were lysed in buffer A containing 0.5% Triton X-100 and centrifuged at 12,000 g for 15 min at 4°C. The following primary antibodies were used to detect total or phosphorylated Akt, S6K, ERK2, JNK and P38 oocyte kinases: anti-Akt1 (C-20) (1∶5000), anti-p70 S6 Kinase alpha (H160) (1∶10000) and anti-ERK2 (1∶10000) were from Santa Cruz Biotechnology; anti-phospho Akt (Thr308) and anti-phospho Akt (Ser 473) (1∶5000) from Upstate Biotechnology; anti-phospho p44/p42 MAPK (ERK1/2) (Thr 202/Tyr 204) (1∶10000), anti-phospho p70 S6 Kinase (Thr 389) (1∶5000) and anti-phospho p38 MAPK (Thr 180/Tyr 182) from Cell Signalling Technology; anti-c-jun N-terminal kinase JNK (1∶10000) from Sigma; anti-active JNK polyclonal antibodies (1∶8000) from Promega; p38 (Xp38γ/SAPK3) antiserum prepared by immunizing rabbits with purifed GST-Xp38γ [44] was kindly provided by Prof. A.R. Nebreda (Barcelona, Spain). Mouse, rabbit or goat Trueblot® secondary antibodies (eBioscience) were used as secondary antibodies and chemoluminescence was revealed using the advanced ECL detection system (Amersham Biosciences).

### Yeast cDNA library screening

Intracellular domains (ICD) of SmVKR1^YYRE^ and SmVKR2^YYRE^ [30] were amplified by PCR using respectively SmVKR1^YYRE^ICDF/SmVKR1^YYRE^ICDR and SmVKR2^YYRE^ICDF/SmVKR2^YYRE^ICDR as primers (Table S2). Insertion of SmVKR1^YYRE^ICD and SmVKR2^YYRE^ICD in pGBKT7 plasmid (Gal4-BD-containing vector) was performed by directional cloning using EcoRI/PstI and BamHI/NcoI restriction sites respectively. Fusion of bait proteins in frame with Gal4-BD was checked by sequencing. Y187 yeasts were transformed by pGBKT7 bait constructs using the Lithium acetate method and plated on selective growth media, as described in the Yeast Protocols Handbook (Clontech). Transformed Y187 cells were then mated with the *S. mansoni* library formed by pGAD (Gal4-AD-containing vector)-transformed AH109 cells, as described before [34]. Diploid yeasts were selected on the quadruple dropout medium, SD-Leu/-Trp/-His/-Ade. Positive clones were submitted to the beta-galactosidase filter assay using X-Gal substrate, following manufacturer's instructions (Yeast Protocols Handbook, Clontech). Confirmed clones were amplified and plasmid DNA was extracted using the NucleoSpin® Plasmid QuickPure kit (Macherey Nagel), after lysis of yeasts with glass beads (Sigma). Plasmid DNA was used to transform chemically competent DH5α bacteria (Invitrogen). Two successive rounds of plating on ampicilline-containing plates were performed to select bacteria containing pGADT7-Rec plasmids. Insert sequences were determined and the nature of preys identified using the BLASTn tool.

### Phylogenetic studies

MEK and PP2C protein sequences were aligned using ClustalW algorithm in the BioEdit v7.1 software, and manually corrected. Maximum likelihood trees were built using MEGA5 [68] under the JTT model, with 1000 bootstrap repetitions.

### *In silico* analyses

Sequences were analyzed using the LASERGENE package (DNAStar, Madison, WI, USA). BLASTn analyses of sequences obtained from the library screening were performed using the NCBI databank http://blast.ncbi.nlm.nih.gov.gate2.inist.fr/Blast.cgi.

## Supporting Information

Figure S1 **Localization of** ***Smvkr1*** **and** ***Smvkr2*** **transcripts in male** ***S. mansoni*** **testes by** ***in situ*** **hybridization.** Transcripts of *Smvkr1* (A) and *Smvkr2* (B) were detected in testes following overexposure to the dye substrate. Scale bar: 50 µm.
(PDF)

Figure S2 **Quantification of** ***Smvkr1*** **and** ***Smvkr2*** **transcripts in ovaries isolated from immature or sexually-mature female parasites.** A) Comparison of the transcription rates of *Smvkr1* and *Smvkr2* in single-sex (ss) and bi-sex (bs) ovaries. The transcription rates of *Smvkr1* were defined with the value 1. Both genes are expressed at the same level in bs ovaries but *Smvkr2* is 2.6-fold more expressed than *Smvkr1* in ss ovaries. B) Comparison of the level of expression of *Smvkr1* and *Smvkr2* in ss versus bs ovaries. Transcription rates of *Smvkr1* and *Smvkr2* in ss ovaries were defined with the value 1. Results were obtained by relative quantification against the actin reference gene (using the ΔΔCt method).
(PDF)

Figure S3 **The JNK pathway activated by SmVKR1 is sensitive to the JNK inhibitor SP600125 but is independent on the activation of MPF.** As in the case of PG or insulin-stimulated *Xenopus* oocytes, phosphorylation of JNK in SmVKR1-expressing oocytes is inhibited by its specific inhibitor SP600125 but not by the addition of purvanalol, the inhibitor of the cyclin-dependent kinase CDK1 that is responsible for MPF activation and meiosis resumption in the oocyte.
(PDF)

Figure S4 **Phylogenetic identification of the SmPP2C isoform XM_002575792.1.** A maximum likelihood tree was generated using MEGA5 under the JTT matrix-based model with 1000 bootstrap repetitions. The phylogenetic identification of the XM_002575792.1 protein was performed using PP2A and PP2C isoforms of the following species: *Apis cerana* (XP_006616109.1), *Ascaris suum* (ERG87858.1, ERG81121.1), *Campotonus floridanus* (EFN65881.1, EFN73519.1, EFN74235.1), *Clonorchis sinensis* (GAA51813.1), *Crassostrea gigas* (EKC41604.1), *Drosophila melanogaster* (NP_476805.1), *Echinococcus granulosus* (CDJ20730.1, EUB56282.1, *Harpegnathos saltator* (EFN87637.1, BAO01182.1), *Homo sapiens* (AAB38020.1, NP_808820.1, NP_640338.2, NP_817092.1, NP_003611.1, *Hymenolepis microstoma* (CDJ07776.1, CDJ13056.1), *Loa loa* (XP_003139223.1), *Marsupenaeus japonicus* (BAO01182.1), *Mus musculus* (NP_058735.1, NP_058735.1, AAG44661.1, AAM14418.1, NP_848841.2, NP_058606.3), *Rattus norvegicus* (XP_003751139.1), *Schistosoma japonicum* (CAX75108.1, AAX28472.2), *Taenia solium* (XP_003377693.1) and *Xenopus laevis* (NP_001116353.1, NP_001085063.1, NP_001080301.1, NP_001085562.1).
(PDF)

Figure S5 **Phylogenetic identification of the SmMkk7 XM_002574592.1.** A maximum likelihood tree was generated using MEGA5 under the JTT matrix-based model with 1000 bootstrap repetitions. The phylogenetic identification the XM_002574592.1 protein was performed using Mkk1, Mkk3, Mkk4, Mkk5, Mkk6 and Mkk7 proteins of the following species: *Aedes Aegypti* (AAQ68075.1), *Aplysia californica* (XP_005108851.1, XP_005098776.1), *Bombyx mori* (NP_001243912.1), *Drosophila melanogaster* (NP_477162.1, AAC46944.1, XP_002032022.1), *Hydra vulgaris* (XP_002162140.2), *Homo sapiens* (NP_002746.1, AAB40652.1, NP_660143.1, NP_002749.2, NP_660186.1), *Mus musculus* (NP_032953.1, CAA63649.1, NP_035970.1, NP_036073.1, AAB81848.1) and *Xenopus laevis* (NP_001080299.1, NP_001084729.1, NP_001079947.1).
(PDF)

Table S1 **Induction of GVBD in** ***Xenopus*** **oocytes expressing SmVKR1 and SmVKR2 by various L-amino acids (each added at 1 mM).** Induction of GVBD by progesterone (PG) in non-injected oocytes is not affected by the presence of L-amino acids.
(PDF)

Table S2 **List of oligonucleotide sequences used as primers.**
(PDF)
