# Correction: Venus Kinase Receptors Control Reproduction in the Platyhelminth Parasite *Schistosoma mansoni*

**DOI:** 10.1371/journal.ppat.1005798

**Published:** 2016-07-25

**Authors:** Mathieu Vanderstraete, Nadège Gouignard, Katia Cailliau, Marion Morel, Steffen Hahnel, Silke Leutner, Svenja Beckmann, Christoph G. Grevelding, Colette Dissous

Due to concerns raised about Fig 4 and Fig 6, the authors would like to publish the raw, uncropped film for Fig 6 as well as an alternate experiment for Fig 4 which verifies the conclusion of the published image.

The authors and editors confirm that these changes do not alter their findings.

## Supporting Information

S1 File Supplementary data. This file includes the alternate experiment for Figure 4.
(PDF)

S2 File Supplementary data. This file includes the original autorad for Figure 6.
(JPG)
