# Supplementary figures and images for: Venus Kinase Receptors Control Reproduction in the Platyhelminth Parasite Schistosoma mansoni
Source: PLoS Pathog. 2014 May 29;10(5):e1004138. doi: 10.1371/journal.ppat.1004138 (PMC4038586; doi:10.1371/journal.ppat.1004138)

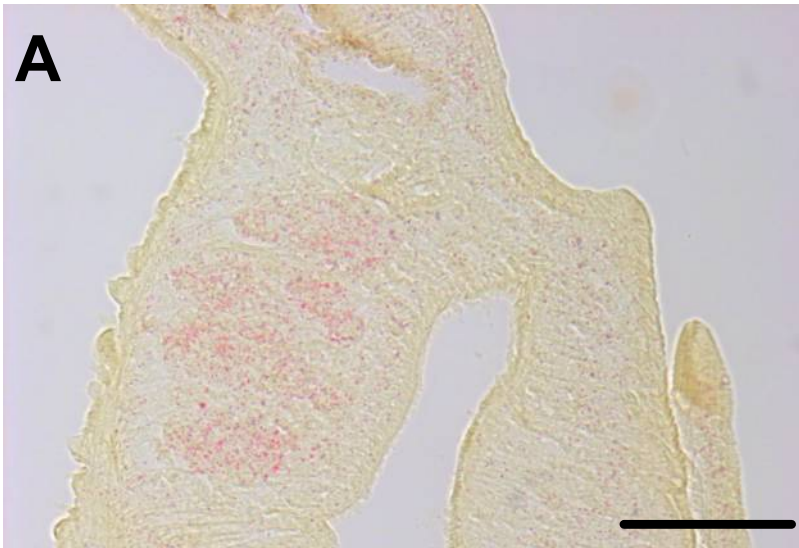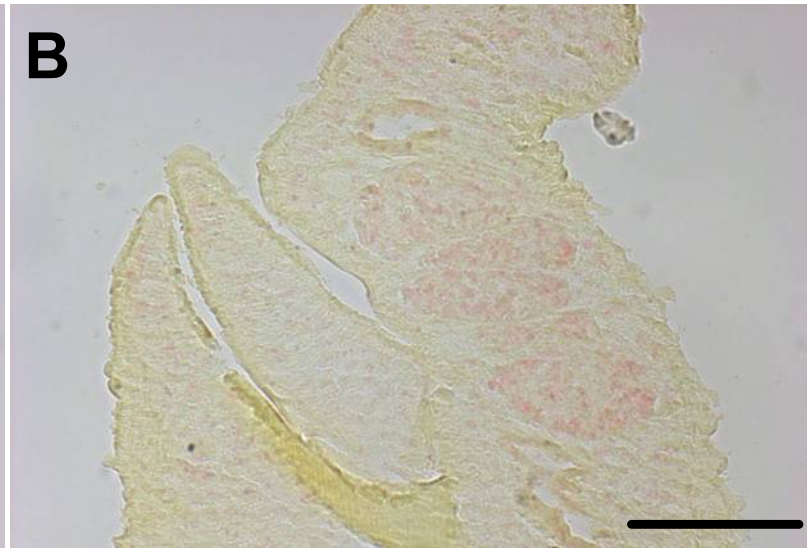

Supplement: Figure S1 — Localization of Smvkr1 and Smvkr2 transcripts in male S. mansoni testes by in situ hybridization. Transcripts of Smvkr1 (A) and Smvkr2 (B) were detected in testes following overexposure to the dye substrate. Scale bar: 50 µm. (PDF) [file ppat.1004138.s001.pdf]

**A**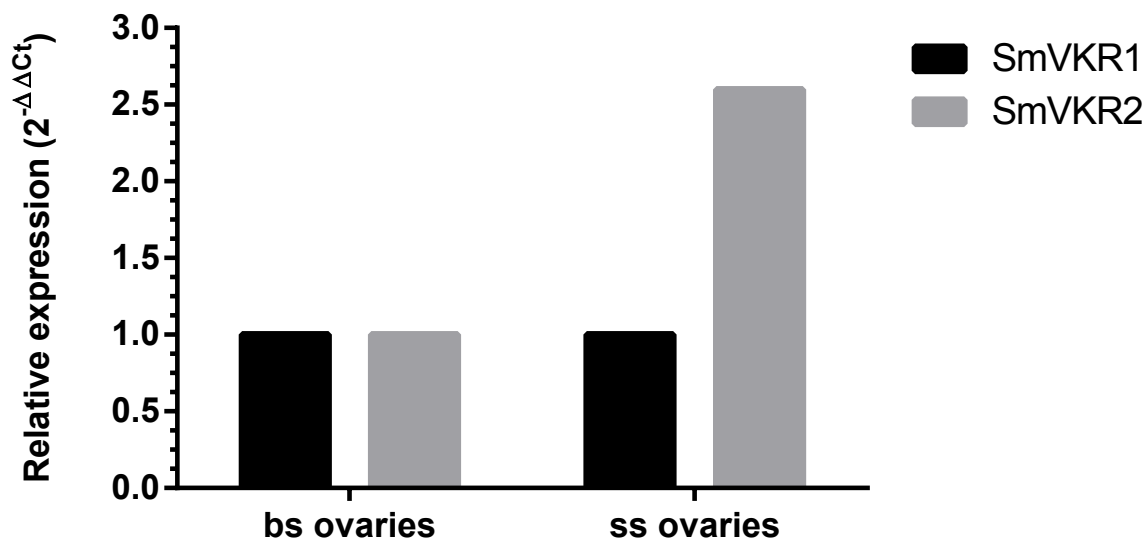**B**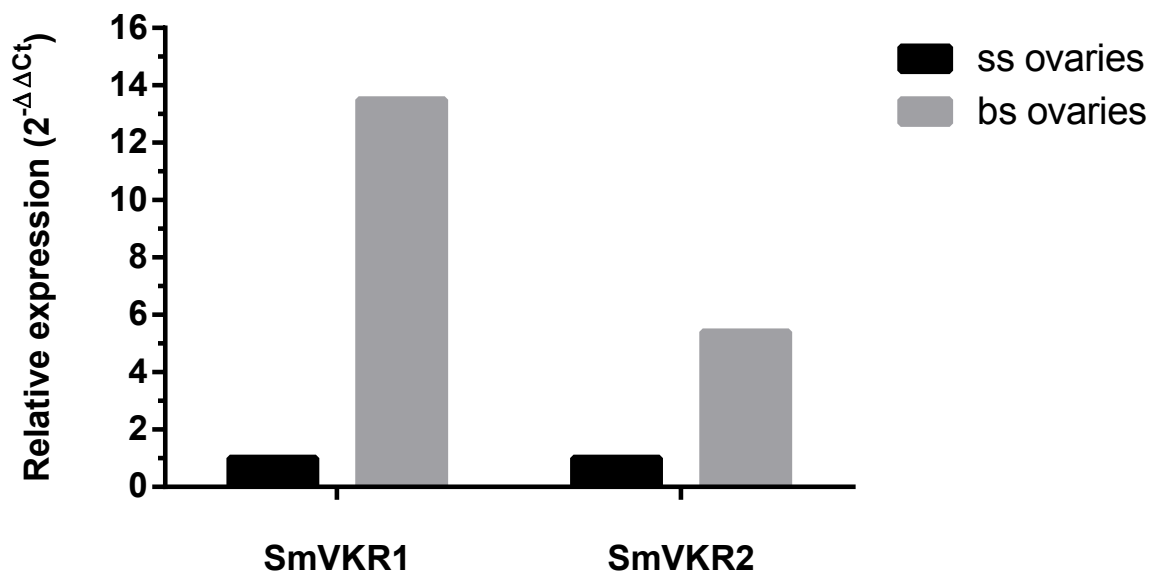

Supplement: Figure S2 — Quantification of Smvkr1 and Smvkr2 transcripts in ovaries isolated from immature or sexually-mature female parasites. A) Comparison of the transcription rates of Smvkr1 and Smvkr2 in single-sex (ss) and bi-sex (bs) ovaries. The transcription rates of Smvkr1 were defined with the value 1. Both genes are expressed at the same level in bs ovaries but Smvkr2 is 2.6-fold more expressed than Smvkr1 in ss ovaries. B) Comparison of the level of expression of Smvkr1 and Smvkr2 in ss versus bs ovaries. Transcription rates of Smvkr1 and Smvkr2 in ss ovaries were defined with the value 1. Results were obtained by relative quantification against the actin reference gene (using the ΔΔCt method). (PDF) [file ppat.1004138.s002.pdf]

|                   |   |   |   |   |   |   |   |   |   |
|-------------------|---|---|---|---|---|---|---|---|---|
| Progesterone      | + | + | + | - | - | - | - | - | - |
| Insuline          | - | - | - | + | + | + | - | - | - |
| SmVKR1            | - | - | - | - | - | - | + | + | + |
| L-Arginine (1μM)  | - | - | - | - | - | - | + | + | + |
| Purvanalol (20μM) | - | + | - | - | + | - | - | + | - |
| SP600125 (100μM)  | - | - | + | - | - | + | - | - | + |

**JNK**

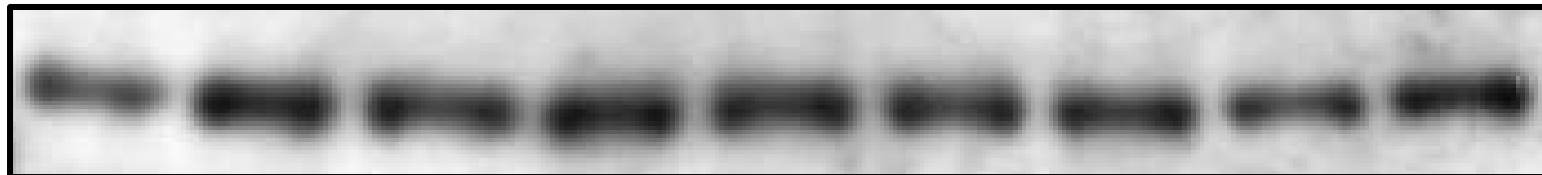

**Phospho-JNK**

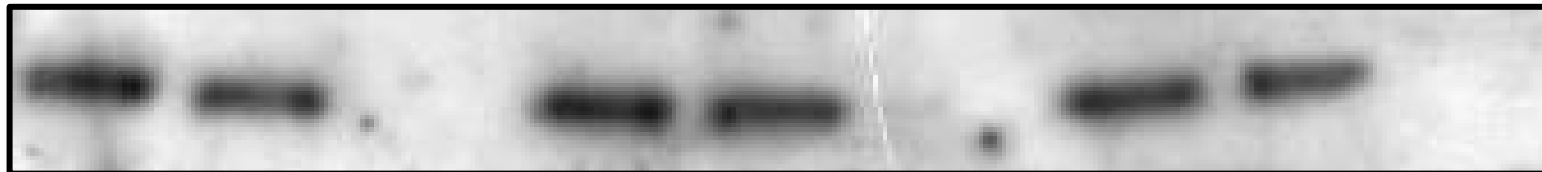

Supplement: Figure S3 — The JNK pathway activated by SmVKR1 is sensitive to the JNK inhibitor SP600125 but is independent on the activation of MPF. As in the case of PG or insulin-stimulated Xenopus oocytes, phosphorylation of JNK in SmVKR1-expressing oocytes is inhibited by its specific inhibitor SP600125 but not by the addition of purvanalol, the inhibitor of the cyclin-dependent kinase CDK1 that is responsible for MPF activation and meiosis resumption in the oocyte. (PDF) [file ppat.1004138.s003.pdf]

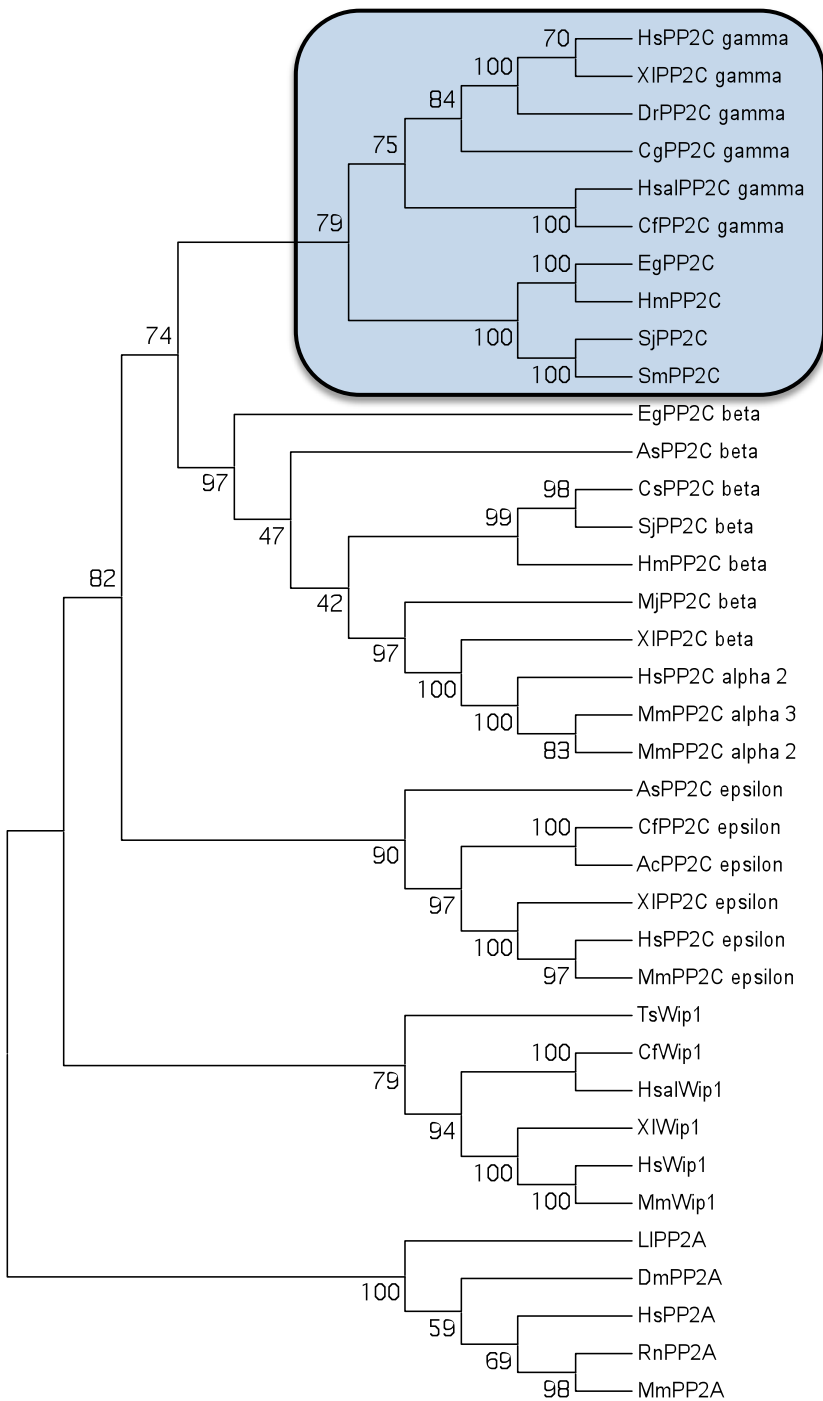

**PPM1G/PP2C  
gamma**

**PPM1B/PP2C  
beta**

**PPM1A/PP2C  
alpha**

**PPM1L/PP2C  
epsilon**

**PPM1D/Wip1**

**outgroup**

Supplement: Figure S4 — Phylogenetic identification of the SmPP2C isoform XM_002575792.1. A maximum likelihood tree was generated using MEGA5 under the JTT matrix-based model with 1000 bootstrap repetitions. The phylogenetic identification of the XM_002575792.1 protein was performed using PP2A and PP2C isoforms of the following species: Apis cerana (XP_006616109.1), Ascaris suum (ERG87858.1, ERG81121.1), Campotonus floridanus (EFN65881.1, EFN73519.1, EFN74235.1), Clonorchis sinensis (GAA51813.1), Crassostrea gigas (EKC41604.1), Drosophila melanogaster (NP_476805.1), Echinococcus granulosus (CDJ20730.1, EUB56282.1, Harpegnathos saltator (EFN87637.1, BAO01182.1), Homo sapiens (AAB38020.1, NP_808820.1, NP_640338.2, NP_817092.1, NP_003611.1, Hymenolepis microstoma (CDJ07776.1, CDJ13056.1), Loa loa (XP_003139223.1), Marsupenaeus japonicus (BAO01182.1), Mus musculus (NP_058735.1, NP_058735.1, AAG44661.1, AAM14418.1, NP_848841.2, NP_058606.3), Rattus norvegicus (XP_003751139.1), Schistosoma japonicum (CAX75108.1, AAX28472.2), Taenia solium (XP_003377693.1) and Xenopus laevis (NP_001116353.1, NP_001085063.1, NP_001080301.1, NP_001085562.1). (PDF) [file ppat.1004138.s004.pdf]

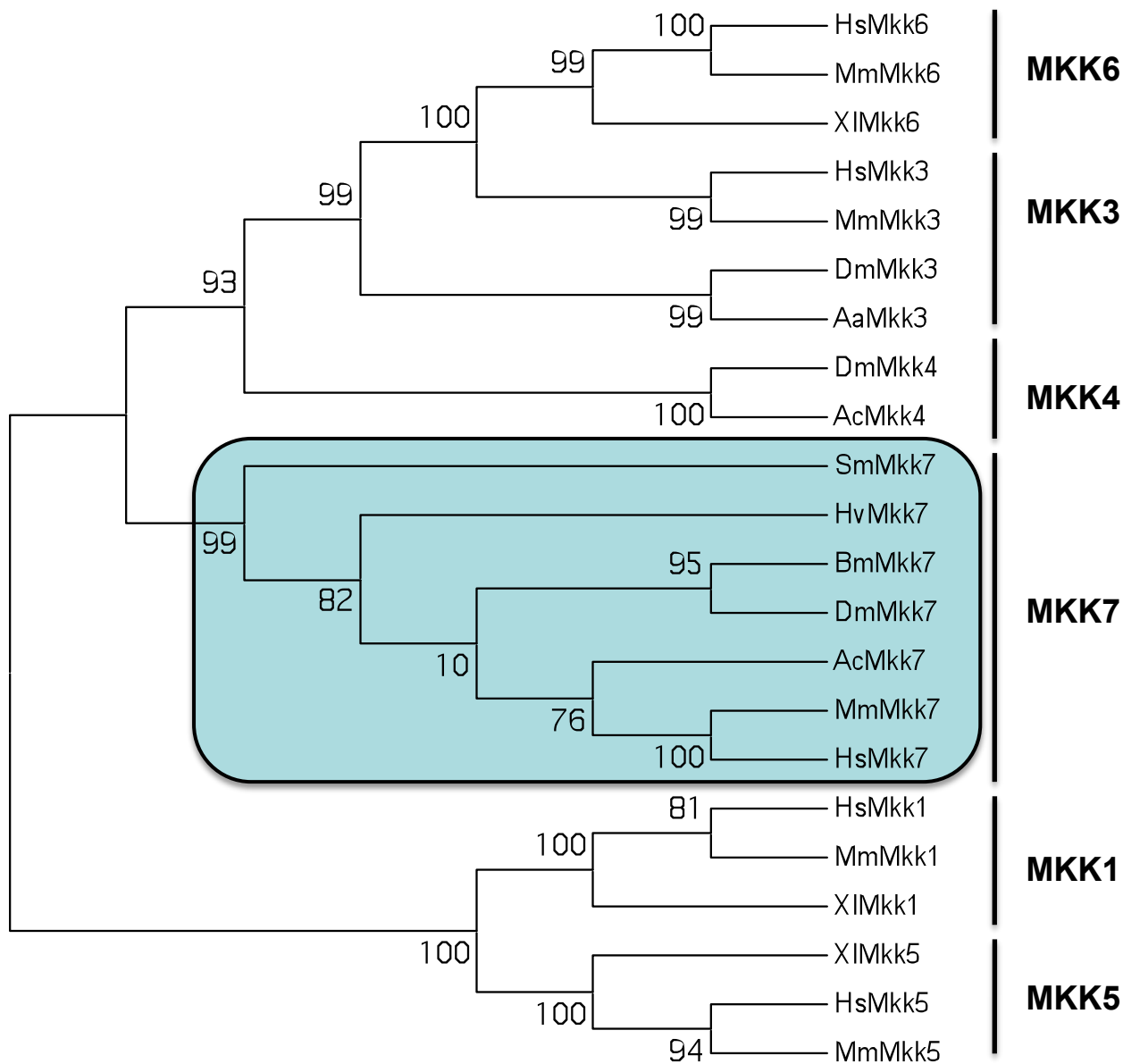

Supplement: Figure S5 — Phylogenetic identification of the SmMkk7 XM_002574592.1. A maximum likelihood tree was generated using MEGA5 under the JTT matrix-based model with 1000 bootstrap repetitions. The phylogenetic identification the XM_002574592.1 protein was performed using Mkk1, Mkk3, Mkk4, Mkk5, Mkk6 and Mkk7 proteins of the following species: Aedes Aegypti (AAQ68075.1), Aplysia californica (XP_005108851.1, XP_005098776.1), Bombyx mori (NP_001243912.1), Drosophila melanogaster (NP_477162.1, AAC46944.1, XP_002032022.1), Hydra vulgaris (XP_002162140.2), Homo sapiens (NP_002746.1, AAB40652.1, NP_660143.1, NP_002749.2, NP_660186.1), Mus musculus (NP_032953.1, CAA63649.1, NP_035970.1, NP_036073.1, AAB81848.1) and Xenopus laevis (NP_001080299.1, NP_001084729.1, NP_001079947.1). (PDF) [file ppat.1004138.s005.pdf]
