# Supplementary material for: Venus Kinase Receptors Control Reproduction in the Platyhelminth Parasite Schistosoma mansoni
Source: PLoS Pathog. 2014 May 29;10(5):e1004138. doi: 10.1371/journal.ppat.1004138 (PMC4038586; doi:10.1371/journal.ppat.1004138)
Supplement: Table S1 — Induction of GVBD in Xenopus oocytes expressing SmVKR1 and SmVKR2 by various L-amino acids (each added at 1 mM). Induction of GVBD by progesterone (PG) in non-injected oocytes is not affected by the presence of L-amino acids. (PDF) [file ppat.1004138.s006.pdf]

AA (1mM)	GVBD (%)		
	PG	SmVKR1	SmVKR2
Ala	100	100	0
Arg	80	100	100
Asn	100	0	0
Asp	100	0	0
Cys	80	80	90
Gln	90	0	0
Glu	100	100	0
Gly	90	80	0
His	100	0	0
Ile	100	0	0
Leu	100	0	0
Lys	100	0	0
Met	90	0	0
Phe	100	0	0
Pro	100	0	0
Ser	100	90	0
Thr	100	100	90
Trp	90	0	80
Tyr	100	0	0
Val	100	0	0
