# Supplementary material for: Venus Kinase Receptors Control Reproduction in the Platyhelminth Parasite Schistosoma mansoni
Source: PLoS Pathog. 2014 May 29;10(5):e1004138. doi: 10.1371/journal.ppat.1004138 (PMC4038586; doi:10.1371/journal.ppat.1004138)
Supplement: Table S2 — List of oligonucleotide sequences used as primers. (PDF) [file ppat.1004138.s007.pdf]

Name	Primer Sequence
SmVKR1HISF	5’-CTGGTGATCCGTCTCCAAGT-3’
SmVKR1HISR	5- AAACGTATGGCCACAGGAAG-3’
SmVKR2HISF	5’-GGACCAACAACAACAATCGTATGATCC-3’
SmVKR2HISR	5’-CGAGGCATATCTAAATCAGCCAATTCTG-3’
SmVKR1RNAiF	5- GGACTCAAATTCACCCACCCCCTCATGTC-3’
SmVKR1RNAiFT7	5’-TAATACGACTCACTATAGGGAGACACCCACCCCCTCATGTC-3’
SmVKR1RNAiR	5’-GATTGACATGACAGGAGATGAAG-3’
SmVKR1RNAiRT7	5’-TAATACGACTCACTATAGGGAGAGACATGACAGGAGATGAAG-3’
SmVKR2RNAiF	5’-GTCTACATACCTGCGAACTACC-3’
SmVKR2RNAiFT7	5’-TAATACGACTCACTATAGGGTACTGTCTACATACCTGCGAACTACC-3’
SmVKR2RNAiR	5’-GACTAACGGTGTCAACAGTTGAGC-3’
SmVKR2RNAiRT7	5’-TAATACGACTCACTATAGGGTACTGACTAACGGTGTCAACAGTTGAGC-3’
SmVKR1qPCRF	5’- AGACATTGATATTCCACCTGCC-3’
SmVKR1qPCRR	5’-AACGCCCATTACGCTTCG-3’
SmVKR2qPCRF	5’- CATTACCTTTTCAACCAACTGTGG-3’
SmVKR2qPCRR	5’-TTCTGATATTATACAAGTGTCC-3’
SmVKR2qPCRF2	5'-CAGATTGCATTCGACCATTCC-3'
SmVKR2qPCRR2	5'-TCGTACGAAAACCACCAACAC-3'
Actinq2F	5'-GGAAGTTCAAGCCCTTGTTG-3'
Actinq2R	5'-TCATCACCGACGTAGCTGTC-3'
SmVKR1S466AF	5’-GATCGCTGCTCTGTGTGCGGATTCAATAGAACC-3’
SmVKR2S410AF	5’-CTATCGCTGCTCTATGCGCAGACTCTATAGAGGCTG-3’
