# Supplementary figures and images for: Correction: Venus Kinase Receptors Control Reproduction in the Platyhelminth Parasite Schistosoma mansoni
Source: PLoS Pathog. 2016 Jul 25;12(7):e1005798. doi: 10.1371/journal.ppat.1005798 (PMC4959732; doi:10.1371/journal.ppat.1005798)

1 November 2011

VK2s | WT S466A S466A  
L-Arg 1µM 1µM 1µM 0

IPV5 | WBV5  
WBPy20

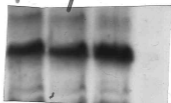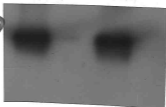

VK2s | WT S410A S410A  
L-Arg 1µM 1µM 1µM 0

IPV5 | WBV5  
WBPy20

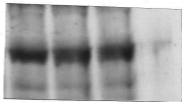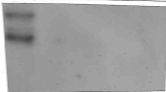

Supplement: S1 File — This file includes the alternate experiment for Figure 4. (PDF) [file ppat.1005798.s001.pdf]

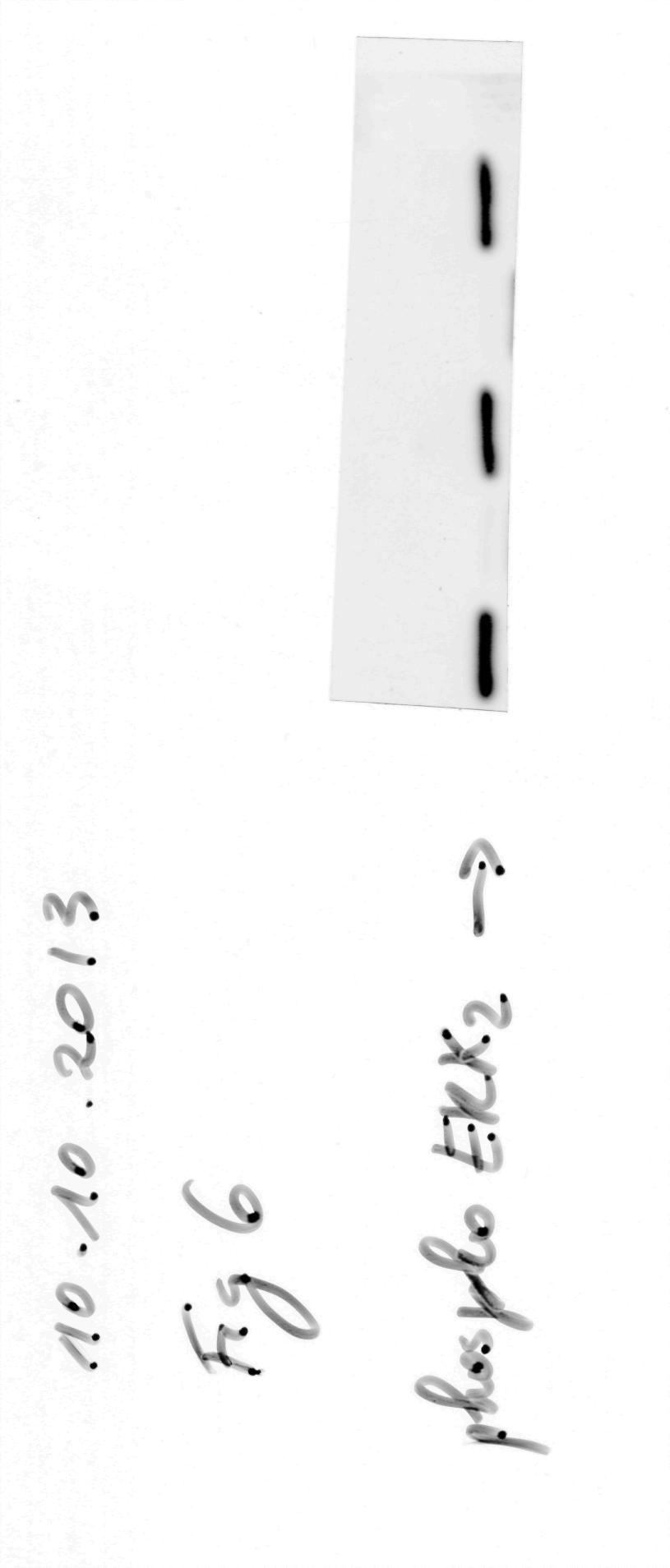

Supplement: S2 File — This file includes the original autorad for Figure 6. (JPG) [file ppat.1005798.s002.jpg]
